# Resolving the lineage relationship between malignant cells and vascular cells in glioblastomas

**DOI:** 10.1093/procel/pwac006

**Published:** 2022-05-18

**Authors:** Fangyu Wang, Xuan Liu, Shaowen Li, Chen Zhao, Yumei Sun, Kuan Tian, Junbao Wang, Wei Li, Lichao Xu, Jing Jing, Juan Wang, Sylvia M Evans, Zhiqiang Li, Ying Liu, Yan Zhou

**Affiliations:** Department of Neurosurgery, Zhongnan hospital of Wuhan University, Frontier Science Center for Immunology and Metabolism, Medical Research Institute, The RNA Institute, College of Life Sciences, Wuhan University, Wuhan 430071, China; Department of Neurosurgery, Zhongnan hospital of Wuhan University, Frontier Science Center for Immunology and Metabolism, Medical Research Institute, The RNA Institute, College of Life Sciences, Wuhan University, Wuhan 430071, China; Department of Neurosurgery, Zhongnan hospital of Wuhan University, Frontier Science Center for Immunology and Metabolism, Medical Research Institute, The RNA Institute, College of Life Sciences, Wuhan University, Wuhan 430071, China; Department of Neurosurgery, Zhongnan hospital of Wuhan University, Frontier Science Center for Immunology and Metabolism, Medical Research Institute, The RNA Institute, College of Life Sciences, Wuhan University, Wuhan 430071, China; Department of Neurosurgery, Zhongnan hospital of Wuhan University, Frontier Science Center for Immunology and Metabolism, Medical Research Institute, The RNA Institute, College of Life Sciences, Wuhan University, Wuhan 430071, China; Department of Neurosurgery, Zhongnan hospital of Wuhan University, Frontier Science Center for Immunology and Metabolism, Medical Research Institute, The RNA Institute, College of Life Sciences, Wuhan University, Wuhan 430071, China; Department of Neurosurgery, Zhongnan hospital of Wuhan University, Frontier Science Center for Immunology and Metabolism, Medical Research Institute, The RNA Institute, College of Life Sciences, Wuhan University, Wuhan 430071, China; Department of Neurosurgery, Zhongnan hospital of Wuhan University, Frontier Science Center for Immunology and Metabolism, Medical Research Institute, The RNA Institute, College of Life Sciences, Wuhan University, Wuhan 430071, China; Department of Neurosurgery, Zhongnan hospital of Wuhan University, Frontier Science Center for Immunology and Metabolism, Medical Research Institute, The RNA Institute, College of Life Sciences, Wuhan University, Wuhan 430071, China; Department of Neurosurgery, Zhongnan hospital of Wuhan University, Frontier Science Center for Immunology and Metabolism, Medical Research Institute, The RNA Institute, College of Life Sciences, Wuhan University, Wuhan 430071, China; Department of Neurology, The Central Hospital of Wuhan, Tongji Medical College, Huazhong University of Science and Technology, Wuhan 430014, China; Skaggs School of Pharmacy, Department of Medicine, Department of Pharmacology, University of California at San Diego, La Jolla, CA 92093, USA; Department of Neurosurgery, Zhongnan hospital of Wuhan University, Frontier Science Center for Immunology and Metabolism, Medical Research Institute, The RNA Institute, College of Life Sciences, Wuhan University, Wuhan 430071, China; Department of Neurosurgery, Zhongnan hospital of Wuhan University, Frontier Science Center for Immunology and Metabolism, Medical Research Institute, The RNA Institute, College of Life Sciences, Wuhan University, Wuhan 430071, China; Department of Neurosurgery, Zhongnan hospital of Wuhan University, Frontier Science Center for Immunology and Metabolism, Medical Research Institute, The RNA Institute, College of Life Sciences, Wuhan University, Wuhan 430071, China

**Keywords:** glioblastoma, mural cells, endothelial cells, trans-differentiation, lineage tracing, single-cell sequencing, copy number variation

## Abstract

Glioblastoma multiforme (GBM), a highly malignant and heterogeneous brain tumor, contains various types of tumor and non-tumor cells. Whether GBM cells can trans-differentiate into non-neural cell types, including mural cells or endothelial cells (ECs), to support tumor growth and invasion remains controversial. Here we generated two genetic GBM models *de novo* in immunocompetent mouse brains, mimicking essential pathological and molecular features of human GBMs. Lineage-tracing and transplantation studies demonstrated that, although blood vessels in GBM brains underwent drastic remodeling, evidence of trans-differentiation of GBM cells into vascular cells was barely detected. Intriguingly, GBM cells could promiscuously express markers for mural cells during gliomagenesis. Furthermore, single-cell RNA sequencing showed that patterns of copy number variations (CNVs) of mural cells and ECs were distinct from those of GBM cells, indicating discrete origins of GBM cells and vascular components. Importantly, single-cell CNV analysis of human GBM specimens also suggested that GBM cells and vascular cells are likely separate lineages. Rather than expansion owing to trans-differentiation, vascular cell expanded by proliferation during tumorigenesis. Therefore, cross-lineage trans-differentiation of GBM cells is very unlikely to occur during gliomagenesis. Our findings advance understanding of cell lineage dynamics during gliomagenesis, and have implications for targeted treatment of GBMs.

## Introduction

The developmental process of multicellular organisms is highly stereotypic, with three germ layers giving rise to distinct cellular progenies that constitute functional tissues and organs. The brain is mostly composed of neural cells that are progenies of the ectoderm, including neurons, astrocytes, ependymal cells, and oligodendrocytes (OLGs; Merkle and Alvarez-Buylla, 2006; Gao et al., 2014). In addition, cellular components of brain vasculature and microglial cells have distinct origins. Mural cells, vascular smooth muscle cells (vSMCs) and pericytes, originate from the neural crest lineage and the mesoderm (Korn et al., 2002; Kurz, 2009; Yamanishi *et al.,* 2012; He and Soriano, 2015), and endothelial cells (ECs) and microglial cells are derivatives of the mesoderm and hematopoietic progenitors in the yolk sac, respectively (Ginhoux et al., 2010). Macrophages, also originating from the yolk sac, can infiltrate into brain parenchyma upon inflammation and gliomagenesis (Poon et al., 2017; Quail and Joyce, 2017).

Glioblastoma multiforme (GBM) are malignant primary brain tumors with properties of high invasiveness and abundant blood supply (Furnari et al., 2007). Deciphering the cellular composition and lineage history of the GBM micro-environment, including ECs, mural cells, and tumor-associated microglia and macrophages (TAMs), has important implications for developing targeted therapies for GBM. GBM cells are thought to originate from mutated neural stem/progenitor cells (Alcantara Llaguno et al., 2009; Liu et al., 2011; Lee et al., 2018; Alcantara Llaguno et al., 2019; Wang et al., 2020). Previous studies indicated that GBM stem cells might give rise to vascular ECs (Ricci-Vitiani et al., 2010; Wang et al., 2010; Soda et al., 2011; Hu et al., 2016; Mei et al., 2017) or mural cells (Scully et al., 2012; Cheng et al., 2013; Zhou et al., 2017) to support tumor growth and invasion. Our earlier studies also found that stem cell markers can label both glioma stem-like cells and mural cells (Zhong et al., 2016; Li et al., 2017), suggesting the possibility of cross-lineage trans-differentiation of glioblastoma cells. Moreover, intra-tumoral trans-differentiation in lung, prostate, brain, and gastric cancers could confer heterogeneity and novel properties on tumors, enabling tumors better adapt to growth and treatment (Murata-Kamiya et al., 2007; Han et al., 2014; Zou et al., 2017; Yao et al., 2020).

Recent advances of single-cell technologies, combined with lineage-tracing analyses, have deep implications for understanding cellular components, cell status and fate transitions during development, tissue homeostasis, regeneration, and pathological conditions (Guimaraes-Camboa et al., 2017; Kester and van Oudenaarden, 2018; Neftel et al., 2019; Bhaduri et al., 2020; Wang et al., 2021). Particularly, genomic signatures such as somatic single-nucleotide variants were recently applied for study lineage history of human cells during development (Bizzotto et al., 2021). Here we combined lineage-tracing approaches with single-cell sequencing to dissect lineage relationships between malignant cells and non-neural cells, especially ECs and mural cells, using both human GBM samples and mouse GBM models. First, genetic lineage-tracing and transplantation studies inferred that GBM cells only extremely rarely gave rise to vascular cells *in vivo*. Intriguingly, however, GBM cells could promiscuously express mural cell markers, including *Tbx18*, during GBM tumorigenesis. We further showed almost non-overlapping copy number variation (CNV) signatures between GBM cells and vascular cells in both mouse and human GBMs. Thus, contradictory to previous opinion, cross-lineage trans-differentiation of GBM cells is uncommon, occurring only in extremely rare events that are more the exception than the norm.

## Results

### Establishing genetic GBM models to trace fates of GBM cells

**Figure 1.**
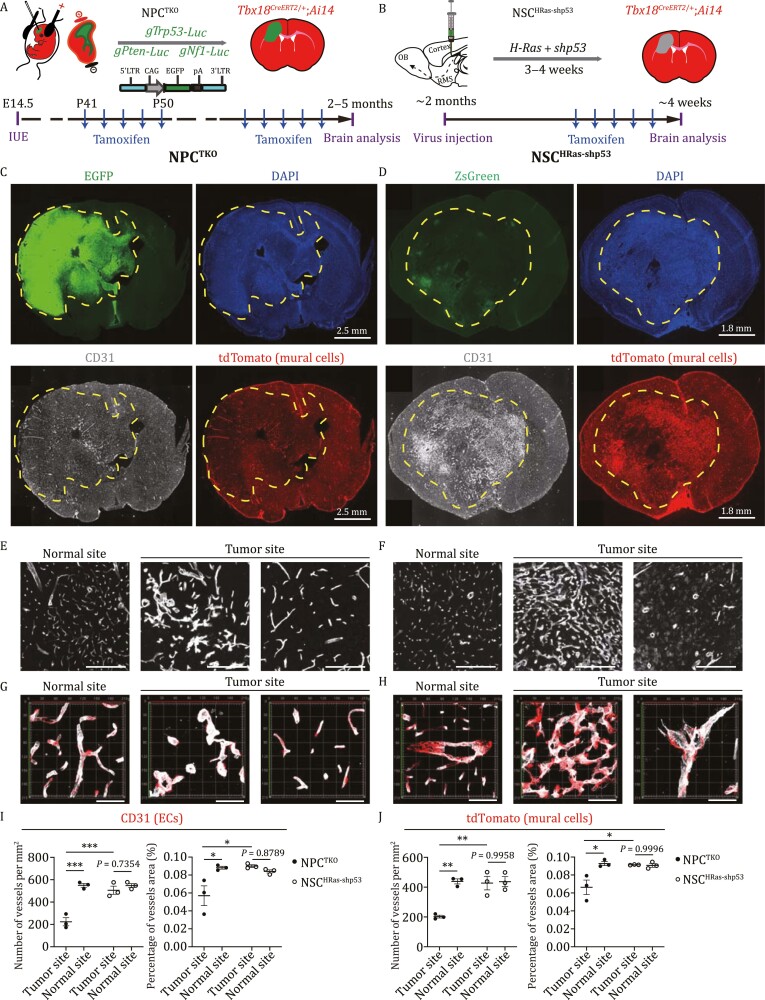
**Gliomagenesis induces widespread alterations of brain vasculature.** (A and B) The schematic illustrations showing *in vivo* gliomagenesis of NPC^TKO^ and NSC^HRas-shp53^ GBMs by (A) *in utero* electroporation and (B) injection of lentiviruses, respectively. (C and D) Representative NPC^TKO^ (C) and NSC^HRas-shp53^ (D) GBM brain sections of *Tbx18*^*CreERT2*/+^;*Ai14* mice were stained with indicated antibodies. CD31 and tdTomato signals depicting ECs and mural cells, respectively. (E–H) Images showing immunofluorescent staining of CD31 (E and F) and CD31/tdTomato (G and H) of NPC^TKO^ (E and G) and NSC^HRas-shp53^ (F and H) GBM brain sections. Scale bars, 200 µm (E and F), 60 μm (G and H). One normal (non-tumor) and two tumor regions of each model were shown. G and H were Z-stack images. Scale bars, 60 μm. (I and J) Comparisons of vessel numbers and area inside and across two GBM models by using CD31 (I) and tdTomato (J) signals, respectively. *n* = 3 for NPC^TKO^ and *n* = 3 for NSC^HRas-shp53^. Data are mean ± SEM. Statistical significance was determined using two-way ANOVA, after checking that our data fitted to a normal distribution (assessed by the Shapiro–Wilk normality test), *P < 0.05; **P < 0.01; ***P < 0.001.

We first utilized genetic mouse models of GBM to investigate whether glioma cells could give rise to non-neural cells. Embryonic neural progenitor cells (NPCs) were transformed into GBM-initiating cells (GICs) by inactivating *Trp53*, *Pten*, and *Nf1*, the three most mutated tumor suppressors in human GBM (Kwon et al., 2008; Chen et al., 2015; Yu et al., 2020). This GBM model was named as NPC^TKO^. Briefly, plasmids co-expressing Cas9, luciferase (Luc) and single-guide RNAs (sgRNAs) targeting *Trp53*, *Pten*, and *Nf1* (Cancer Genome Atlas Research, 2008; Chen et al., 2012), along with a lineage-labeling cassette that stably expresses enhanced green fluorescent protein (EGFP) or Discosoma red fluorescent protein (DsRed) in progeny of GICs (Cheng et al., 2016), were *in utero* electroporated into neocortical neural precursors at embryonic day 14.5 (Fig. 1A). Notably, the genome-editing and lineage-labeling cassette was incorporated into embryonic NPCs via the piggyBac transposase system (Fig. S1A). Adult mice were screened for brain tumors by Luc-mediated imaging analysis. The tumor mass was extensively labeled with cytosolic EGFP or DsRed depending on the lineage-labeling cassette used. Intracranial tumors showed typical features of diffusely infiltrating high-grade gliomas: prominent tumor mass with high proliferation evidenced by pleomorphic and mitotic nuclei, widespread Ki67^+^ cells, indistinct tumor borders, pseudopalisading necrosis, and dilated vasculature (Fig. S1B–F). GBM sections were stained with markers for neurons (NeuN), OLGs (MBP), astrocytes (GFAP), oligodendrocyte precursor cells (OPC, OLIG2), and neural stem cells (SOX2). Around 50%–80% of GBM cells expressed OLIG2, and 10% expressed GFAP (Fig. S1G), indicating OPC features for most GBM cells, aligning with cellular properties observed in OPC-like GBMs (Liu et al., 2011; Wang et al., 2020; Yu et al., 2020). Moreover, approximately 60%–80% of tumor cells also expressed SOX2, suggesting their undifferentiated status (Fig. S1G). EGFP^+^ or DsRed^+^ tumor cells were sorted out from four samples for transcriptome analyses. Gene Set Enrichment Analysis (GSEA) was used to examine the relatedness of NPC^TKO^ GBMs to molecular signatures of human GBMs. Among the four NPC^TKO^ GBM cells examined, three of them displayed molecular features of mixed The Cancer Genome Atlas proneural (TCGA-PN) and mesenchymal (MES) GBMs, with one bearing predominantly the TCGA-PN feature (Verhaak et al., 2010; Wang et al., 2017; Fig. S1M). Therefore, genetic inactivation of three tumor-suppressor genes in embryonic neural precursor cells (NPC^TKO^) leads to tumorigenesis of TCGA-MES and -PN GBMs in adult mice.

In parallel, we established another GBM genetic model by transforming adult neural precursors into GICs *in situ* (Fig. 1B). Lentiviruses co-expressing *Trp53* short hairpin RNAs (shRNAs) and human constitutively active *HRAS*^*G12V*^ oncogene were injected into the ventricular-subventricular zone of adult C57BL/6 mice (NSC^HRas-shp53^), which developed high-grade glioma with typical histopathological features of GBM within 3–4 weeks after lentiviral injection (Fig. S1I; Marumoto et al., 2009; Neftel et al., 2019). Single-cell RNA sequencing (scRNA-seq) analysis of mouse NSC^HRas-shp53^ GBMs showed that each tumor contained three cellular states identified in human GBM, i.e. OPC-like, astrocyte-like (AC-like), and MES-like (Neftel et al., 2019). Interestingly, NSC^HRas-shp53^ GBMs contained significantly fewer OLIG2-expressing cells than NPC^TKO^ GBMs (Fig. S1J–L). RNA-seq transcriptome analyses indicated all three NSC^HRas-shp53^ GBMs molecularly recapitulate the TCGA-MES subtype of human GBMs (Fig. S1M). Moreover, like other genetic GBM models, both NPC^TKO^ and NSC^HRas-shp53^ tumors displayed mixed transcriptome signatures of OLGs, astrocytes, and OPCs but not neurons (Fig. S1N; Cahoy et al., 2008); and were associated with the OLG lineage-associated GBM subtype (Type 2) (Fig. S1O; Alcantara Llaguno et al., 2015; Wang et al., 2020).

### GBM tumorigenesis induces widespread alterations of brain vasculature

We next examined how the two types of GBMs affected vasculature structures in the brain. NPC^TKO^ and NSC^HRas-shp53^ GBMs were generated *de novo* in *Tbx18*^*CreERT2*/+^;*Ai14* brains in which mural cells were genetically labeled upon tamoxifen administration. In line with previous studies, 99.8% of pericytes (CD146^+^PDGFRB^+^) and 99.6% of ACTA2-expressing vSMCs were co-labeled with *Tbx18*-driven tdTomato in wild-type *Tbx18*^*CreERT2*/+^;*Ai14* brains, reconfirming the reliability of *Tbx18* in labeling mural cells (Fig. S2A). Consistently, mural cells of NPC^TKO^ and NSC^HRas-shp53^ GBMs were extensively labeled with *Tbx18*-driven tdTomato and overlaid CD31-labeled ECs (Fig. 1G and 1H). In both models, the CD31 signals of ECs were enhanced, and many tumor vessels were dilated, stiffened, concentrated, or highly disorganized (Fig. 1C–H). To our surprise, in NPC^TKO^ GBMs there were fewer vessels and decreased vessel coverage in tumor regions relative to non-tumor sites, with vessels exhibiting glomeruli-like structures. In contrast, in NSC^HRas-shp53^ GBMs, vessels were greatly dilated and interwoven into net-like structures, with increased vessel density and vessel area when compared to vessels in NPC^TKO^ GBMs (Fig. 1E–J). Since NPC^TKO^ GBMs contained significantly more OLIG2^+^ cells than NSC^HRas-shp53^ GBMs (Fig. S1L), these observations are in line with a previous study showing that OLIG2-positive GBMs had almost normal vasculature, whereas OLIG2-negative GBMs promote angiogenesis and blood–brain barrier breakdown (Griveau et al., 2018).

### Minimal trans-differentiation of GBM cells into mural cells

**Figure 2.**
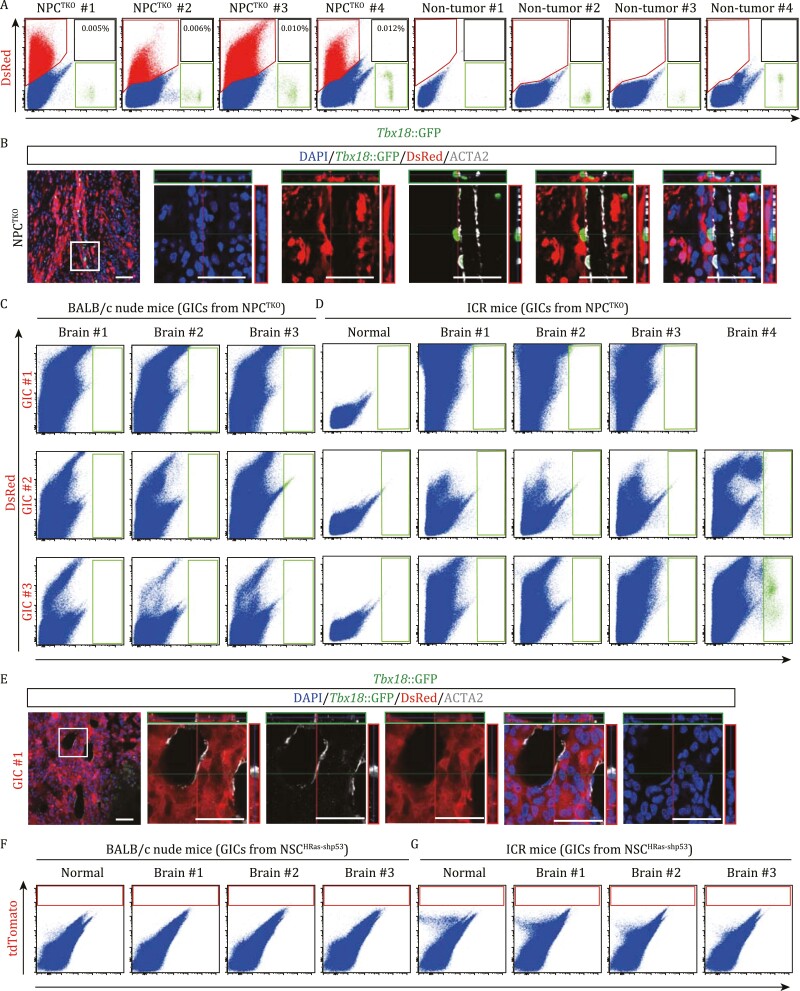
**Glioblastoma cells scarcely gave rise to mural cells in genetic GBM brains, but could promiscuously express *Tbx18* upon allografting.** (A) Flow cytometry assays for four NPC^TKO^ GBM and four non-tumor brains (*Tbx18*^*H2B-GFP*/+^) indicating minimal co-labeling of DsRed^+^ GBM cells with *Tbx18*-expressing mural cells. Percentages of GFP-expressing GBM cells were shown. (B) Z-stack images (boxed area in the left panel) of the section of a NPC^TKO^(*Tbx18*^*H2B-GFP*/+^) GBM brain. Scale bars, 50 μm. (C and D) Flowcytometry assays of BALB/c nude (C) and ICR (D) brains allografted by three GIC lines derived from NPC^TKO^(*Tbx18*^*H2B-GFP*/+^) GBMs. DsRed signals represent GBM cells. (E) BALB/c nude mice were intracranially allografted with GICs derived from a NPC^TKO^(*Tbx18*^*H2B-GFP*/+^) GBM. Representative immunofluorescent (left) and Z-stack images showing expressions of *Tbx18*::H2B-GFP and spatial relationships of allografted DsRed-expressing GICs with ACTA2^+^ mural cells. Scale bars, 50 μm. (F and G) Flow cytometry assays of BALB/c nude (F) and ICR (G) brains allografted by GICs derived from a NSC^HRas-shp53^(*Tbx18*^*CreERT2*/+^;*Ai14*) GBM.

We next explored the possibility of trans-differentiation of GBM cells into mural cells. Pregnant *Tbx18*^*H2B*-*GFP*/+^ mice were electroporated to allow us to examine fate choices of DsRed-labeled malignant cells in NPC^TKO^ GBMs. In line with previous studies, almost all mural cells, i.e., 99.4% of vSMCs and 99.7% of pericytes, were genetically labeled by *Tbx18*-driven nuclear GFP (H2B-GFP) in *Tbx18*^*H2B*-*GFP*/+^ brains (Fig. S2A; Guimaraes-Camboa et al., 2017). If GICs could differentiate into mural cells, we expected to observe DsRed^+^/GFP^+^ double-positive cells, because most GBM cells stably expressed DsRed. Flow cytometry analyses showed that DsRed^+^ GBM cells and *Tbx18*::H2B-GFP^+^ mural cells were separate populations, with only very few double-labeled cells detected (Fig. 2A), the latter of which could be rare trans-differentiation events or misexpression of *Tbx18* by some GBM cells.

In parallel studies, tumor sections were immunostained for *Tbx18*::H2B-GFP and smooth muscle actin alpha 2 (ACTA2), a marker for most vSMCs. Confocal imaging and 3D reconstruction demonstrated that, although many DsRed^+^ GBM cells were in close proximity to blood vessels, no overlap between GBM cells and the ACTA2 signal or *Tbx18*::H2B-GFP could be detected (Figs. 2B and S2B; Video S1).

### Negligible differentiation of GBM stem-like cells into mural cells following intracranial grafting

Previous studies suggested that GICs with stem-like features could mostly recapitulate pathological and molecular features of primary GBMs upon intracranial transplantation. In addition, mouse GICs derived from the two GBM models could propagate tumors in both immunocompromised and immunocompetent environments, which facilitated additional lineage-tracing studies. Tumor cells derived from NPC^TKO^(*Tbx18*^*H2B-GFP*/+^) GBMs were *in vitro* cultured in serum-free conditions to propagate GICs (Ignatova et al., 2002; Pollard et al., 2009) followed by fluorescence-activated cell sorting (FACS) to enrich DsRed^+^ GICs. We next grafted DsRed^+^ GICs intracranially into nude and ICR mice to produce allograft tumors. No DsRed^+^/*Tbx18*::H2B-GFP^+^ or DsRed^+^/ACTA2^+^ double-positive cells were observed in sections of intracranial tumors by IF staining (Figs. 2E and S2C). By flow cytometry, although no *Tbx18*::H2B-GFP^+^ double-positive cells were detected in 9 nude brain grafts or in 11 outbred ICR grafts, two ICR grafts contained a very small portion of *Tbx18*::H2B-GFP cells: of 2.8 million cells analyzed, only 365 and 3523 *Tbx18*::H2B-GFP cells were detected, respectively, in the two ICR grafts (Fig. 2C and 2D). However, these *Tbx18*::H2B-GFP cells did not express ACTA2 and located away from ACTA2-labeled vessels (Fig. S2F). We then stained sections of these two ICR brains to address whether these *Tbx18*::H2B-GFP cells represented fully trans-differentiated mural cells or whether they were GBM cells that misexpressed *Tbx18*. IF data showed that ~98% of *Tbx18*::H2B-GFP cells co-expressed GBM markers SOX2 and/or OLIG2, and exhibited a clustered distribution consistent with clonal propagation, suggesting that *Tbx18* was being misexpressed by a subset of GBM cells that retained expression of GBM markers (Fig. S2D and S2E).

In addition, GICs derived from an NSC^HRas-shp53^(*Tbx18*^*CreERT2*/+^;*Ai14*) GBM sample were implanted into brains of BALB/c nude and ICR mice. Tamoxifen was injected once per week for the first 2 weeks and three times in the third week (Fig. S2F). Therefore, cells that expressed *Tbx18* would be labeled by tdTomato. Flow cytometry analyses did not detect tdTomato^+^ cells (progeny of *Tbx18-*expressing cells) in either nude or ICR GBM grafts (Fig. 2F and 2G). Moreover, IF staining showed that the few rarely detected tdTomato^+^ cells in these grafts were located distantly from CD31^+^ vessels and co-expressed SOX2 and/or OLIG2, indicating their GBM cell properties (Fig. S2G and S2H). Next, we transplanted human GBM stem-like cells labeled with ZsGreen into nude mouse brains (Li et al., 2017), but did not observe ZsGreen^+^ACTA2^+^ cells in all brain sections (Fig. S3). Altogether, these findings demonstrated that GICs were barely able to differentiate into mural cells in allografted or xenografted animals, although rarely a small percentage of GBM cells expressed markers for mural cells during gliomagenesis.

### GBM cells failed to trans-differentiate into ECs

To examine the potential trans-differentiation of GBM cells into hematopoietic or endothelial lineages, we performed lineage-tracing studies by generating NPC^TKO^ and NSC^HRas-shp53^ GBMs in *Tek*-Cre;*Ai14* brains, in which 99.8% of ECs (Kisanuki et al., 2001) and 90.1% of TAMs (Gomez Perdiguero et al., 2015) were genetically labeled with cytosolic tdTomato (Fig. S4A). TAMs are derived from brain-resident microglia and infiltrated macrophages and express Iba1 (Poon et al., 2017; Quail and Joyce, 2017).

**Figure 3.**
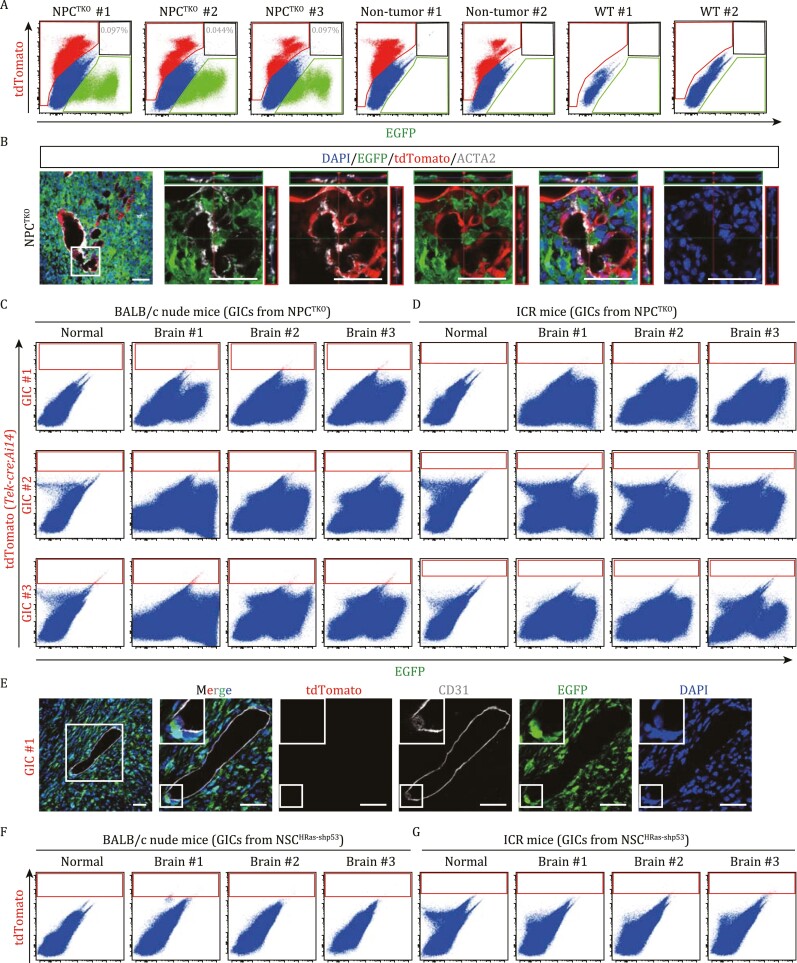
**Glioblastoma cells did not give rise to ECs and TAMs in genetic glioblastoma brains.** (A) Flow cytometry assays for three NPC^TKO^ (*Tek-Cre*;*Ai14*) GBM brains and two *Tek-Cre;Ai14* non-tumor brains indicating minimal co-labeling of EGFP^+^ glioblastoma cells with tdTomato-expressing ECs and TAMs. Percentages of tdTomato-expressing GBM cells were shown. Two wild-type (WT) brains were used for gating. (B) Z-stack images (boxed area in the left panel) of the section of a NPC^TKO^ (*Tek-Cre*;*Ai14*) GBM brain showing spatial relationship between tumor cells (EGFP^+^), ECs (tdTomato^+^), and mural cells (ACTA2^+^). Scale bars, 50 μm. (C and D) Flow cytometry assays of BALB/c nude (C) and ICR (D) brains allografted by three GIC lines derived from NPC^TKO^ (*Tek-Cre*;*Ai14*) GBMs. EGFP signals represent GBM cells. (E) BALB/c nude brains were allografted with GICs derived from a NPC^TKO^ (*Tek-Cre*;*Ai14*) GBM. Scale bars, 50 μm. Representative images showing immunofluorescent staining of brain sections using indicated antibodies. The boxed area was enlarged on right panels. (F and G) Flow cytometry assays of BALB/c nude (F) and ICR (G) brains allografted by a GIC lines derived from a NSC^HRas-shp53^ GBM sample.

At postnatal days 60–150, GBM brains were minced and digested into single-cell suspensions for flow cytometry analyses. Akin to findings showed in Fig. 2A, EGFP^+^ GBM cells and tdTomato^+^ cells labeled by *Tek*-Cre were distinct populations, with only a small fraction of double-labeled cells detected (Fig. 3A), likely owing to misexpression of *Tek* by a few GBM cells during tumorigenesis. Interestingly, vessel walls were largely intact and did not show evidence of GBM cells invading or being incorporated into vascular structures, with numerous Iba1^+^ TAMs aggregated around tumor vessels (Fig. S4D). Many tumor vessels were fully or partially covered with ACTA2-expressing mural cells (Fig. 3B). Importantly, although many EGFP^+^ tumor cells of NPC^TKO^ GBMs were in close proximity to blood vessels, no overlap of EGFP with the tdTomato signal was observed, arguing against cross-lineage differentiation of EGFP-expressing GBM cells into tdTomato-labeled endothelial or microglia/macrophage lineages (Figs. 3B and S4E; Video S2).

### Absence of EC differentiation by GBM stem-like cells following intracranial grafting

Next, EGFP^+^ GICs derived from three NPC^TKO^(*Tek-Cre*;*Ai14)* GBMs were grafted into brains of immunocompromised BALB/c nude mice or immunocompetent ICR mice to produce allograft tumors. If GICs could differentiate into ECs, we expected to observe EGFP^+^/tdTomato^+^ double-positive cells. However, in flow cytometry experiments, we did not detect EGFP^+^/tdTomato^+^ cells from grafted tumors (Fig. 3C and 3D). Consistent with these results, only a few EGFP^+^/tdTomato^+^ cells were visualized in sections of intracranial tumors; however, these double-labeled cells lacked EC morphology and did not express CD31 (Figs. 3E and S4F).

To ask whether GBM cells derived from NSC^HRas-shp53^ tumors could produce ECs, we also implanted GICs enriched from NSC^HRas-shp53^(*Tek-Cre*;*Ai14*) GBMs into brains of BALB/c nude and ICR mice. In line with findings using NPC^TKO^ GICs, by flow cytometry we did not detect tdTomato^+^ cells (progeny of *Tek-*expressing cells) from grafted tumors derived from NSC^HRas-shp53^ GICs (Fig. 3F and 3G). Rare tdTomato^+^ cells could be seen in grafted brains; however, these cells were located distantly from vessels and did not express CD31 (Fig. S4G), indicating misexpression of *Tek* rather than trans-differentiation. Thus, GICs did not give rise to ECs *in vivo* under either immunocompromised or immunocompetent conditions in our experimental settings.

### scRNA-seq of GBM cells distinguishes tumor from non-tumor cell types

**Figure 4.**
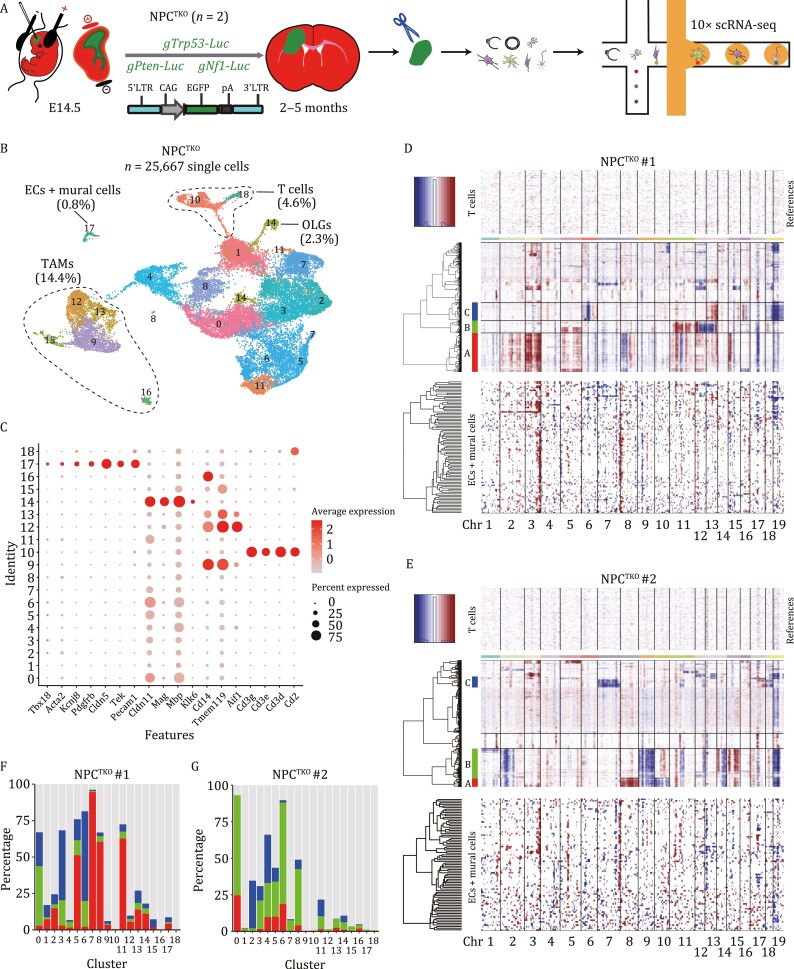
**CNV patterns and distributions of vascular cells and malignant cells in NPC** ^**TKO**^ **GBM samples.** (A) The schematic illustration showing *in vivo* gliomagenesis of NPC^TKO^ GBMs by *in utero* electroporation followed by scRNA-seq. (B) UMAP plots visualizing cell clustering of NPC^TKO^ GBM samples. Clusters and ratios of ECs and mural cells, T cells, TAMs, and OLGs were indicated. (C) Dot plots showing expression levels of selected marker genes across clusters of NPC^TKO^ GBMs. (D and E) CNV patterns of two NPC^TKO^ GBMs are shown, with red and blue colors indicating amplifications and deletions, respectively. Using the inferCNV algorithm, inference of chromosomal CNVs was based on average relative expression in windows of 100 analyzed genes. Rows correspond to cells; non-malignant T cells that lack CNVs are shown at the top, followed by other cells ordered by overall CNV patterns. Clusters of malignant cells were marked as colored bars. CNV patterns of ECs and mural cells were shown at the bottom. (F and G) Bar plots showing percentages of malignant cells in each cluster of two NPC^TKO^ GBM samples. Cells in Cluster 17 were designated as vascular cells.

We next deployed scRNA-seq to characterize distinct cell types within GBM samples followed by analyses of lineage relationships between malignant cells and vascular cells. Two GBM specimens from the NPC^TKO^ model, one of each being generated in either *Tek*-Cre;*Ai14* or *Tbx18*^*CreERT2*/+^;*Ai14* brains, were dissected and enzymatically digested into single-cell suspensions, then subjected to 10× Genomics scRNA-seq. A total of 25,667 single cells were successfully sequenced (Fig. 4A).

Uniform Manifold Approximation and Projection (UMAP) was employed for dimensionality reduction to cluster cells, which stratified cells into 18 principal cell clusters (Figs. 4B and S5A; Table S1). Well-established marker genes were then mapped onto the UMAP representation to designate cell types. Specifically, vascular cells (*Pecam1*, *Cldn5*, and *Tek* for ECs; *Pdgfrb*, *Kcnj8*, *Acta2*, and *Tbx18* for mural cells) (Fig. S5B and S5C), TAMs (*Cd14*, *Aif1*, and *Tmem119*), OLGs (*Klk6*, *Mag*, *Mbp*, and *Cldn11*), and T lymphocytes (*Cd2*, *Cd3d*, *Cd3e*, and *Cd3g*) were respectively clustered into distinct groups. Notably, clusters of ECs and mural cells were either non-separable or close to each other, indicating their lineage kinship. Putative tumor cells (Fig. 4B and 4C) widely expressed neural stem cell (NSC) markers (*Sox2*, *Nestin*, and *Olig2*) and *Pdpn*, a marker indicating the mesenchymal-like state of GBM cells (Hara et al., 2021; Fig. S5E). As expected, expression of *Luciferase* and *Egfp* could be mostly detected in cells showing NSC features (Fig. S5D). However, interestingly, expression of markers for ECs or mural cells was not confined to vascular cell clusters, but could be also detected, although at comparably moderate levels, in other cell types. This was particularly true for *Cspg4* (*Ng2*), a marker also labeling OPCs, which was highly expressed in GBM cells. Consistently, although expression of *tdTomato* was predominant in vascular cells and TAMs, it could be also detected in other cell clusters, demonstrating aberrant expression of vascular cell markers during GBM tumorigenesis (Fig. S5D). On the other hand, expression of NSC markers could be detected at relatively moderate levels in non-neural cells (Fig. S5E), echoing a recent scRNA-seq study of human GBMs (Bhaduri et al., 2020).

In addition, a substantial portion of tumor cells and non-tumor cells expressed *Mki67*, indicative of their proliferative status (Fig. S5E). In summary, analyses of scRNA-seq data of NPC^TKO^ GBMs supported our lineage-tracing analyses by showing that although vascular cells and TAMs clustered in groups clearly distinct from those of malignant cells, some GBM cells promiscuously expressed markers of other lineages, thus emphasizing the need to exercise extreme caution before assigning cell-type identity by using a few individual marker(s).

### CNVs of vascular cells are distinct from those of GBM cells

Cells with common ancestors bear similar genetic variations such as CNVs, and recent studies have analyzed lineage relationships according to CNVs (Patel et al., 2014; Venteicher et al., 2017). To further explore the lineage relationship between tumor cells and non-tumor cells, we therefore utilized scRNA-seq data to compare CNV signatures of GBM cells with those of vascular cells. Our assumption was that if ECs and mural cells arise by trans-differentiation from GBM cells, they should share common CNVs with tumor cells. We performed CNV analyses in NPC^TKO^ GBM samples, utilizing CNVs of T cells as the reference (Fig. 4D and 4E). Using the inferCNV algorithm, CNVs were estimated by sorting the analyzed genes by their chromosomal location and applying a moving average to the relative expression values, with a sliding window of 100 genes within each chromosome. Hierarchical clustering grouped the rest single cells into multiple cell groups based on their CNV properties. Three groups of malignant cells (Group A/B/C) could be identified from each of the two NPC^TKO^ GBM specimens based on their prominent CNV characteristics (Tables S2 and S3), including genomic deletions and amplifications, which showed intra-tumoral and inter-tumoral heterogeneity. We found that CNV patterns of vascular cells were distinct from those of malignant cells. Cells recognized as malignant were then assigned into cellular clusters previously grouped according to their transcriptome signatures (Fig. 4B and 4C). Not surprisingly, clusters 0, 3, 4, 5, 6, 7, 8, and 11 of the two NPC^TKO^ GBMs contained significant portions of malignant cells. More than 90% of vascular cells from the two NPC^TKO^ GBM samples (cluster 17: 84/92 of NPC^TKO^ #1 and 80/81 of NPC^TKO^ #2) were designated as non-malignant cells (Fig. 4F and 4G). For the few malignant cells that were clustered into vascular cells, we reasoned that these cells might display some transcriptome features of vascular cells. Alternatively, some vascular cells could acquire a certain CNV characteristics of malignant cells due to clonal expansion (Zhou et al., 2020). In addition, the copyKAT (copynumber karyotyping of tumors) algorithm was applied to assign CNVs for individual cells (Gao et al., 2021), which also designated most vascular cells as diploid non-malignant cells: 85/92 for NPC^TKO^ #1 and 80/81 for NPC^TKO^ #2.

Next, we superimposed CNV signals and correlations between vascular cells and T cells on those of each batch of malignant cells. “CNV signal” reflects the overall extent of CNVs of each cell, defined as the mean of the squares of CNV values across the genome. “CNV correlation” refers to the correlation between the CNV profile of each cell and the average CNV profile of all malignant cells from the corresponding tumor cluster. Of the three clusters of malignant cells in the two NPC^TKO^ GBM samples, CNV properties for vascular cells and T cells were largely distinct from malignant cells, supporting distinct lineage origins for these cell types (Fig. S6A–L). Of note, CNVs of NSC^HRas-shp53^ GBM cells were less drastic than those of NPC^TKO^ GBM cells, probably due to different onco-drivers and/or the shorter gliomagenesis period for NSC^HRas-shp53^ GBMs (data not shown).

### CNVs of vascular cells are distinct from those of malignant cells in human GBM samples

**Figure 5.**
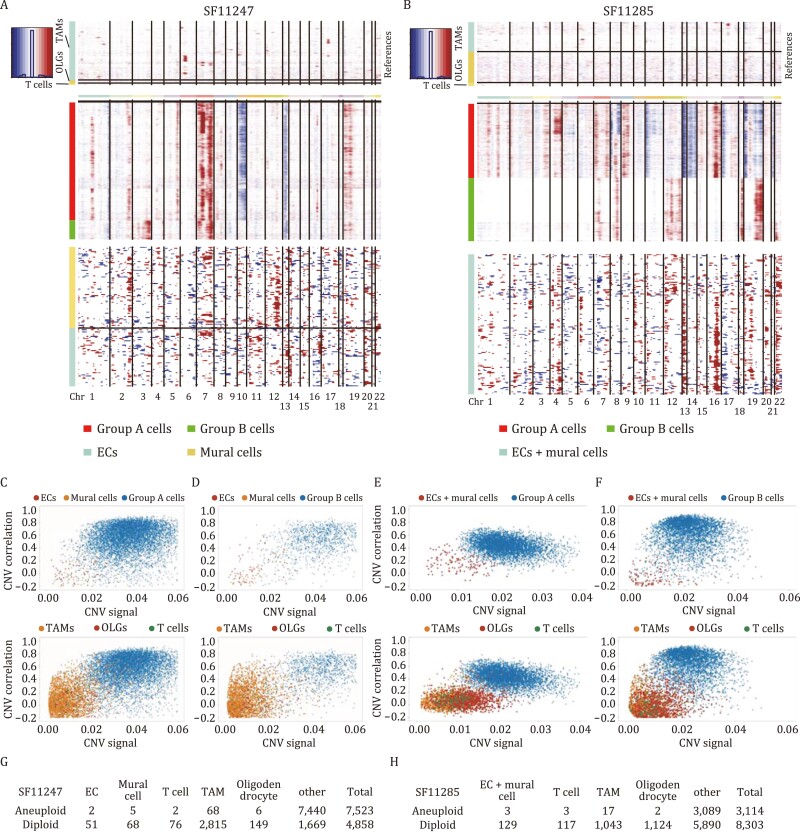
**CNV patterns were separated between human GBM cells and vascular cells.** (A and B) CNV patterns of two human GBM samples—SF11247 (A) and SF11285 (B) were shown. Using the inferCNV algorithm, inference of chromosomal CNVs was based on average relative expression in windows of 100 analyzed genes. Rows correspond to cells; non-malignant TAMs, OLGs and T cells that lack CNVs are shown at the top, followed by other cells ordered by overall CNV patterns. CNV patterns of ECs and mural cells were shown at the bottom. (C–F) Plots depicting CNV signals, and correlations of vascular cells (top), TAMs, OLGs, and T cells (bottom), with different groups of malignant cells—SF11247 (C and D) and SF11285 (E and F). (G and H) Numbers of indicated cell types designated as aneuploid malignant and diploid normal cells in SF11247 (G) and SF11285 (H) GBMs were calculated using the copyKAT algorithm.

To investigate the possibility that ECs and mural cells of human GBMs could derive from malignant cells, we analyzed CNV features of human GBM samples. To this end, high-quality scRNA-seq data of two GBM samples—SF11247 and SF11285—generated by Bhaduri et al. was analyzed (Bhaduri et al., 2020). For sample SF11247, 12 398 cells were grouped into 18 clusters, with ECs and mural cells in close-related but distinct clusters (Fig. S7A, S7C, and S7E; Table S4). For sample SF11285, 11 433 cells were grouped into 14 clusters, with ECs and mural cells in the same cluster (Fig. S7B, S7D, and S7F; Table S5). Similar to findings with mouse GBM samples, expression of most vascular cell markers could be also detected in other cell types (Fig. S7E and S7F). CNV analyses were performed utilizing scRNA-seq data, designating CNVs of T cells, TAMs, and OLGs as the reference. We empirically split GBM cells of each sample into two groups according to their CNV properties (Fig. 5A and 5B). For example, Group A cells of SF11247 showed GBM hallmarks of chromosome 7 gain and chromosome 10 loss; whereas Group B cells of SF11247 displayed gain of chromosome 3 and 10, as well as loss of chromosome 13 (Fig. 5A). As to SF11285, Group A cells contained widespread amplifications and deletions; but Group B cells had prominent amplifications of chromosome 7, 8, 12, 18, and 19 (Fig. 5B). Consistent with data acquired from mouse genetic models, CNV patterns of vascular cells were largely distinct from those of tumor cells. Intriguingly, although some vascular cells shared a few common CNV signatures with malignant cells, including amplification of chromosome 16, CNVs unique to vascular cells such as the amplification of chromosome 13 could be detected in SF11285 (Fig. 5B), suggesting potential clonal expansion following this chromosomal abnormality, similar to findings in a recent report which found that some stromal cells, including immune cells, fibroblasts, and ECs, of human colorectal cancer carried genomic alterations with distinct features probably due to clonal expansion (Zhou et al., 2020).

We then calculated and compared CNV signals and CNV correlations with malignant cells (Neftel et al., 2019). In both tumors, similar to non-tumor TAMs, OLGs, and T cells, most vascular cells had lower CNV signals and were less correlated with GBM cells (Fig. 5C–F), thus forming distinct groups. Consistently, the copyKAT algorithm also assigned the majority of vascular cells as diploid non-malignant cells: for GBM SF11247, ECs—49/51, mural cells—63/68; for GBM SF11285, 126/129. Notably, a small ratio of T cells, TAMs, and OLGs were also assigned as aneuploid cells (Fig. 5G and 5H; Table S6). Thus, CNV properties of vascular cells were distinct from malignant cells in both mouse and human GBMs, strongly suggesting that vascular cells were derived from lineage(s) other than GBM cells.

### ECs and mural cells propagate upon GBM tumorigenesis

**Figure 6.**
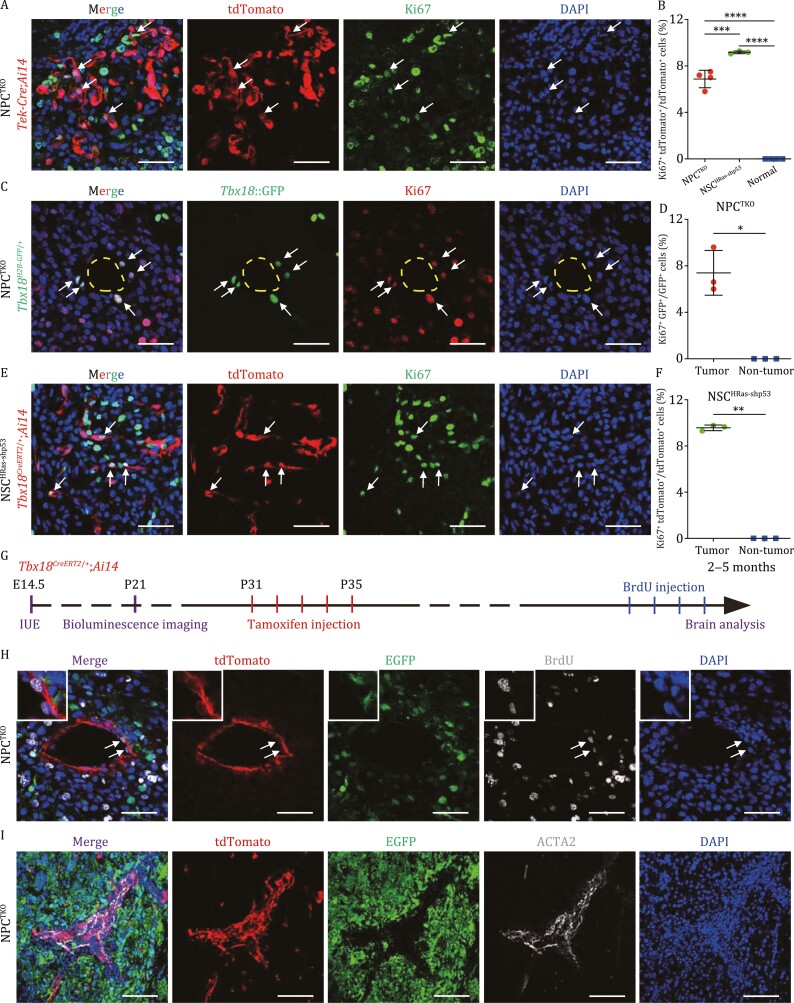
**ECs and mural cells self-propagate during gliomagenesis.** (A–F) GBM brain sections from NPC^TKO^(*Tek-Cre*;*Ai14*), NPC^TKO^(*Tbx18*^*H2B-GFP*/+^), and NSC^HRas-shp53^(*Tbx18*-CreERT2;*Ai14*) mice were quenched with EGFP and DsRed signals for tumor cells followed by immunostainings for tdTomato (A and E), *Tbx18*::GFP (C), and Ki67 (A, C, and E). Ki67^+^ ECs and mural cells were indicated with arrows. Percentages of Ki67^+^tdTomato^+^/tdTomato^+^ (B and F) and Ki67^+^GFP^+^/GFP^+^ (D) cells were quantified. *n* = 3 animals for each genotype. (G) The timeline for *in vivo* gliomagenesis by *in utero* electroporation (IUE), live tumor detection, and phenotypic analyses in NPC^TKO^(*Tbx18*^*CreERT2*/+^;*Ai14*) mouse brains. (H and I) Representative brain sections from (G) were immunostained with BrdU, with BrdU^+^ mural cells indicated with white arrows and enlarged at top-left corners (H). Representative GBM brain sections from (G) were immunostained with ACTA2 (I). Scale bars, 50 μm. Data are mean ± SEM. Statistical significance was determined using one-way ANOVA followed by Tukey’s *post hoc* test (B) or an unpaired two-tailed Student’s *t*-test (D and F), *P < 0.05; **P < 0.01; ***P < 0.001; ****P < 0.0001.

Single-cell sequencing had demonstrated the presence of proliferating cells, as marked by expression of *Ki67*, in both tumor cells and vascular cells (Figs S5D, S7E, and S7F). To further investigate cell types that were proliferating, tumor sections of NPC^TKO^ and NSC^HRas-shp53^ brains from genetically labeled animals were co-immunostained with Ki67. Results demonstrated that in NPC^TKO^ GBM brains, ~7% of tdTomato^+^ cells in *Tek-Cre*;*Ai14* mice, and ~7% of *Tbx18*::H2B-GFP^+^ cells were Ki67^+^ proliferative cells, whereas matched brain regions on the contralateral side had no marker^+^/Ki67^+^ cells (Fig. 6A–D). NSC^HRas-shp53^ GBM brains contained ~9% of tdTomato^+^Ki67^+^ cells in both *Tek-Cre*;*Ai14* mice and *Tbx18*^*CreERT2*/+^;*Ai14* mice (Fig. 6B, 6E, and 6F). Next, resident mural cells and their progeny were labeled by injecting tamoxifen into *Tbx18*^*CreERT2*/+^;*Ai14* mice at P31–P35, when gliomagenesis of NPC^TKO^ GBMs is at an early stage. Brain tumors were acquired from 2- to 5-month-old mice for immunofluorescence analyses, with BrdU administrated for four consecutive days prior to sacrifice (Fig. 6G). Results showed that ~10% of genetically labeled ECs and mural cells in NPC^TKO^ GBM brains had incorporated BrdU (Figs. 6H, S8A and S8C). Similar results were seen in NSC^HRas-shp53^ GBM brains (Fig. S8D and S8E). Therefore, resident mural cells and ECs were activated to proliferate upon gliomagenesis. In concert with these findings, we found ~7.5% of CD31^+^ ECs and ~5% of ACTA2^+^ vSMCs were proliferative in human GBM specimens, but not in non-tumor sites (Fig. S8F–I). Finally, all ACTA2^+^ mural cells in GBM mouse brains were progeny of *Tbx18*-CreERT2;*Ai14* cells in NPC^TKO^ GBM brains (Fig. 6I). Altogether, our findings indicate that amplification of major vascular cell types in GBM tissue is a result of self-propagation rather than their being derived by trans-differentiation of GBM cells.

## Discussion

In the current study, we attempted to resolve the lineage relationship between GBM cells and non-neural cells including ECs, mural cells, and TAMs by combining genetic lineage-tracing studies with CNV comparisons. The human and mouse GBM samples analyzed in current study showed that ECs and mural cells likely belong to lineages separate from GBM cells, and that reprogramming of GBM cells into vascular cells is very unlikely, echoing a previous study indicating absence of trans-differentiation of hEGFRvIII^+^ GBM cells into ECs (Griveau et al., 2018).

Two genetic GBM models were generated *de novo* in immunocompetent mouse brains, which recapitulate key pathological and molecular features of human GBMs. We then applied lineage-tracing studies of NPC^TKO^ and NSC^HRas-shp53^ GBM cells in three genetic models that, respectively, labeled ECs and TAMs, or mural cells, which included *de novo* tumorigenesis and intracranial transplantation of GBM stem-like cells in both immunocompromised and immunocompetent mice. Minimal co-labeled GBM cells and ECs/hematopoietic cells were observed either by flow cytometry or by extensive examination of tissue sections. Intriguingly, a few *Tbx18*-expressing NPC^TKO^ GBM cells were detected in a few primary and implanted tumors, which might support trans-differentiation of GBM cells into mural cells. However, in the two transplanted NPC^TKO^ GBMs where some malignant cells co-expressed *Tbx18*-driving GFP, these cells mostly aggregated as clones and expressed SOX2 and/or OLIG2, but did not express ACTA2, indicating their GBM cell identity. We did not observe marker-misexpressing cells that showed mural cell morphology and/or had incorporated into tumor vasculature, suggesting that these marker-misexpressing cells were unlikely to be at a transitional state between GBM cells and mural cells.

We further compared CNV patterns between malignant cells and vascular cells in NPC^TKO^ GBMs. Clusters of vascular cells (ECs and mural cells) were well separated from malignant cells according to their transcriptome characteristics. Importantly, vascular cells in NPC^TKO^ GBMs exhibit CNV features distinct from malignant cells, arguing against trans-differentiation of GBM cells into ECs and mural cells. Further analyses of single-cell sequencing data derived from two human GBM samples also showed that CNV characteristics of ECs and mural cells were quite different from those of malignant GBM cells, supporting a non-GBM cell-of-origin for vascular cells.

Single-cell transcriptome studies also suggested that many markers that have been used to label mural cells in past studies, including *Acta2*, *Pdgfrb*, *Tbx18*, and *Cspg4*, could be expressed by cells beyond vascular cell clusters. The promiscuous expression of *Tbx18* during gliomagenesis was further supported by dispersed tdTomato expression in *Tbx18*^*CreERT2*^;*Ai14* GBMs. Therefore, misexpression of mural cell markers by some GBM cells, probably due to disorganized epigenomes and signaling cascades, could lead to the conclusion that the cross-lineage trans-differentiation of GBM cells might occur. However, these “misexpressing” cells do not meet transcriptome and functional criteria for ECs and mural cells, as they also expressed markers of NSCs without displaying typical morphology of vascular cells. Interestingly, some vascular cells carried common CNV signatures, indicating their clonal expansion during gliomagenesis. A recent study found that significantly more stromal cells, including fibroblasts, ECs, and immune cells, of colorectal cancer carried CNVs than those in adjacent normal tissues (Zhou et al., 2020). We also showed that a significant portion of vascular cells were actively cycling in GBM tissues but not in non-tumor tissues. Therefore, tumorigenesis could promote proliferation of stromal cells, and stromal cells that bear CNV features might acquire clonal advantage over those without CNVs. Future studies could apply extensive bioinformatic analyses including CellPhoneDB followed by experimental validations to dissect how GBM cells and vascular cells communicate with each other (Efremova et al., 2020).

Our studies further propose potential explanations as to why previous studies have suggested cross-lineage trans-differentiation of GBM cells. Human GBMs might contain immunocompetent environment analogous to our tumorigenesis and allograft models, which could facilitate propagation of “marker-misexpressing” cells. Additionally, previous studies have largely relied on immunofluorescence staining using molecular markers for mural cells including NG2 and PDGFRB, which are also expressed in both neural and non-neural cell types and might lead to false conclusions (Ishii et al., 2008; Cheng et al., 2013; Dimou and Gallo, 2015). Finally, many GBM cells are tightly associated with blood vessels; thus, GBM cells that misexpress a subset of vascular lineage markers could be mistakenly identified as trans-differentiated cells.

Forced expression of so-called master transcriptional regulators (MRs) along with proper micro-environmental cues is required for trans-differentiation events to occur. For example, short-term ETV2 expression and TGFβ inhibition with constitutive ERG1/FLI1 co-expression reprograms mature amniotic cells into vascular ECs with clinical-scale expansion potential (Ginsberg et al., 2012). Similarly, expression of MYOCD reprograms human skin fibroblasts into vSMCs (Bersini et al., 2020). Nonetheless, *in vivo* reprogramming, even among the neural lineage, by manipulating MRs remains controversial, probably due to leaky expression of reporter genes and lack of stringent lineage-tracing and single-cell sequencing methods (Wang et al., 2021). From this perspective, reprogramming of GBM cells into vascular cells is likely a rare, if not totally absent, event.

Interestingly, we noticed a few key disparities between the two genetic models of GBMs. The NPC^TKO^ GBMs have mixed molecular features of TCGA-MES and -PN, whereas NSC^HRas-shp53^ GBMs molecularly recapitulate the TCGA-MES subtype of human GBMs. NPC^TKO^ GBM cells contain more prominent genetic alterations compared to NSC^HRas-shp53^ GBMs (data not shown), probably because embryonic neural precursors are at the apex of neurodevelopment hierarchy and longer tumorigenic period for NPC^TKO^ GBMs. We would like to point out that although loss-of-function mutations of three tumor-suppressor genes were introduced at embryonic stages to generate NPC^TKO^ GBMs, those tumors by no means resemble human pediatric high-grade gliomas, which often carry mutations distinct from adult GBMs (Schwartzentruber et al., 2012; Wu et al., 2012). In addition, there was substantial vascular remodeling and increased proliferation of mural cells and ECs in GBM brains, which could be either direct or adaptive responses to GBM formation. Intriguingly, the two genetic GBM models displayed distinct vascular patterns, possibly reflecting their cellular composition and properties such as the relative proportion of OLIG2^+^ cells. Indeed, there was less vessel coverage and fewer vessels in NPC^TKO^ GBMs than NSC^HRas-shp53^ GBMs, mirroring a previous study showing OLIG2^+^ gliomas had largely normal vasculature, but that vessel density, vessel size, and the vascular and luminal areas within OLIG2^−^ gliomas were significantly increased (Griveau et al., 2018).

Here we did not observe cross-lineage trans-differentiation of GBM cells into vascular cells through careful lineage-tracing and single-cell analyses. We cannot exclude, however, given the highly inter-tumoral and intra-tumoral heterogeneity of GBMs, the possibility that a rare number of GBM cells might gain some molecular and functional features of vascular cells. Lineage-tracing studies indeed showed a very small portion of GBM cells misexpressed markers for vascular cells; and a few vascular cells of the NPC^TKO^ GBM were designated as malignant cells based on their CNV properties. Therefore, complementing genetic lineage-tracing experiments with single-cell sequencing could more stringently discern lineage relationships. Future studies should apply more lineage-tracing studies using other markers of mural cells or ECs. Moreover, single-cell sequencing and CNV analyses followed by vascular cell enrichment using additional GBM samples could further improve the resolution in differentiating lineages.

In summary, our findings advance understanding of the lineage potential of GBM cells and might provide insights into targeted treatments for GBM patients.

## Materials and methods

### Key resources table

**Table:** 

Reagent or resource	Source	Identifier
Antibodies		
Ki67	Abcam	Cat# ab15580, RRID: AB_443209
BrdU	Abcam	Cat# ab6326, RRID: AB_305426
SOX2	Millipore	Cat# Ab5603, RRID: AB_2286686
NeuN	Abcam	Cat# ab177487, RRID: AB_2532109
TUJ1	Sigma-Aldrich	Cat# T8660, RRID: AB_477590
GFAP	Agilent	Cat# Z0334, RRID: AB_10013382
MBP	Covance	Cat# SMI-99P, RRID: AB_10120129
OLIG2	Millipore	Cat# AB9610, RRID: AB_570666
OLIG2	Millipore	Cat# MABN50, RRID: AB_10807410
IBA1	Abcam	Cat# ab5076, RRID: AB_2224402
CD31	Abcam	Cat# ab24590, RRID: AB_448167
CD31	R&D	Cat# AF3628, RRID: AB_2161028
ACTA2	Abcam	Cat# ab5694, RRID: AB_2223021
GFP	Abcam	Cat# ab13970, RRID: AB_300798
PTEN	Cell Signaling Technology	Cat# 9552, RRID: AB_10694066
PDGFRB	Santa Cruz Biotechnology	Cat# sc-432, RRID: AB_631068
ACTB	ABclonal	Cat# AC026, RRID: AB_2768234
CD146	BioLegend	Cat# 134701, RRID: AB_1732002
Bacterial and virus strains		
*E*.* coli* DH5α	TransGen	CD201-01
*E*.* coli* Stbl3	TransGen	CD521-01
Biological samples		
Patient-derived GBM tissues	This paper	N/A
Chemicals, peptides, and recombinant proteins		
DMEM/F12	Gibco	Cat# C11330500B1
N-2	Gibco	Cat# 17502048
B-27	Gibco	Cat# 17504044
hFGF2	Gibco	Cat# PHG0261
hEGF	Gibco	Cat# PHG0311
Penicillin-streptomycin	Gibco	Cat# 15140122
Recombinant human TGF-β1	PeproTech	Cat# 100-21
FBS	Lonsera	Cat# S711-001S
DNase I	Fermentas	Cat# EN0521
Papain	Sangon Biotech	Cat# A003124
Accutase	Gibco	Cat# A1110501
Laminin	Sigma-Aldrich	Cat# L2020-1MG
Tamoxifen	Sigma-Aldrich	Cat# T5648-1G
Sunflower seed oil from Helianthus annuus	Sigma-Aldrich	Cat# S5007-250ML
Experimental models: cell lines		
mGIC-DsRed #1	This paper	N/A
mGIC-DsRed #2	This paper	N/A
mGIC-DsRed #3	This paper	N/A
mGIC-EGFP	This paper	N/A
Experimental models: organisms/strains		
Mouse: *Tek-Cre*:* B6.Cg-Tg(Tek-cre)1Ywa*/*J*	The Jackson Laboratory	JAX: 008863
Mouse: *Ai14*:* B6.Cg-Gt(ROSA)26Sor*^*tm14*(*CAG-tdTomato*)*Hze*^/*J*	The Jackson Laboratory	JAX: 007914
Mouse: *Tbx18*^*H2B-GFP*/+^: *Tbx18*^*tm1.1Sev*^	(Cai et al., 2008)	MGI#: 5529155
Mouse: *Tbx18*^*CreERT2*/+^:* Tbx18*^*tm3.1*(*cre*/*ERT2*)*Sev*^/*J*	(Guimaraes-Camboa et al., 2017)	JAX: 031520
Recombinant DNA		
*pPB-sgTrp53-spCas9-luc*	This paper	N/A
*pPB-sgPten-spCas9-luc*	This paper	N/A
*pPB-sgNf1-spCas9-luc*	This paper	N/A
*pPB-EGFP*	This paper	N/A
*pPB-DsRed*	This paper	N/A
Software and algorithms		
FlowJo	FlowJo X 10.0.7r2	
ImageJ	NIH	
GraphPad Prism	GraphPad Software	

### Vector construction

Oligonucleotides coding for guide RNAs targeting *Trp53, Pten*, and *Nf1* have been described previously (Zuckermann et al., 2015). These oligonucleotides were constructed and cloned into the pPB-sgRNA-SpCas9 (Cheng et al., 2016). For the Bioluminescence detection, the EGFP cassette in the pPB-sgRNA-SpCas9 described above was replaced with Luc cassette from pGL3 (Promega). pPB-EGFP and pPB-DsRed were constructed from the pPB-mU6pro vector. The mU6-EGFP cassette in the pPB-mU6pro vector was replaced with CAG-DsRed (from pCALNL-DsRed; #13769) to construct the pPB-DsRed. Then, the DsRed cassette in pPB-DsRed was replaced with EGFP cassette (from pEGFP-C2) to construct pPB-EGFP.

### Animals

*Tbx18* ^*H2B-GFP/+*^ mice (Cai et al., 2008; Guimaraes-Camboa et al., 2017) were maintained on mixed C57BL/6 and CD-1 background. *Tek-Cre*, *Tbx18*^*CreERT2/+*^ (Guimaraes-Camboa et al., 2017) and *Ai14* mice were maintained on the C57BL/6 background. The *Ai14* reporter mice (007914, The Jackson Laboratory) were crossed with *Tek-Cre* (008863, The Jackson Laboratory) and *Tbx18*^*CreERT2/+*^ to genetically lineage trace the progenies of ECs and TAMs (*Tek-Cre*), and mural cells (*Tbx18*^*CreERT2/+*^), respectively. Genotypes of all mice were determined using PCR analyses of tail or toe genomic DNA with appropriate primers. Both male and female mice were used for all experiments without preference. Mice were housed in certified specific-pathogen-free facilities. All animal procedures were approved by the Laboratory Animal Welfare and Ethics Committee of the College of Life Science and Medical Research Institute, Wuhan University.

### *In utero* electroporation

All pregnant mice (E14.5) were deeply anesthetized with pentobarbital sodium (70 mg/kg) for *in utero* electroporation. Surgical operation and electroporation-based gene transfer was performed as follows. After injecting 1–2 µL supercoiled plasmids (5 μg/μL in ddH_2_O) into telencephalic vesicles of E14.5 embryos, electric square pulses were generated using CUY21VIVO-SQ (BEX) and delivered into dorsal forebrains using forceps-like electrodes (35 mv, 50 ms-on, 950 ms-off, 5 pulses). The uteri were then carefully put back into the abdominal cavity and incisions were sutured. The whole procedure was complete within 30 min. Mice were warmed on heating pad until they woke up and given analgesia treatment (Ibuprofen) in drinking water.

### Intracranial injection of lentivirus

Lentiviruses were stereotactically injected into the subependymal ventricular zone of 8-week-old *Tek-Cre*;*Ai14* and *Tbx18*^*CreERT2*/+^;*Ai14* mice. Lentiviruses (1 × 10^6^ IU) suspended in 1.5 µL PBS were loaded on the syringe, and injected slowly (0.2 µL/min) using the following coordinates: 1.0 mm anterior, 1.0 mm lateral, and 2.3 mm dorsal to the bregma. Upon completing injection, the needle was left in place for 5 min, then withdrawn slowly to reduce virus reflux in 2 min.

### Bioluminescence imaging

The *in utero* electroporation-operated embryos were allowed to survive until P21–P30 and were subjected to bioluminescence imaging of Luc activity. Luc signals were captured using the IVIS Lumina II (Caliper Life Sciences). Mice were injected with 30 mg of D-Luciferin, Potassium Salt (Yeasen Biotech) intraperitoneally 8 min before imaging. Images were acquired and radiance was determined within mouse heads. Only mice whose brain carried Luc signals were selected for further analysis. At postnatal days 60–150, all selected mice were subjected to bioluminescence imaging again to make sure tumors were big enough for experiments.

### Tamoxifen administration

To efficiently induce CreER-dependent recombination, tamoxifen (150 mg/kg of body weight, T5648, Sigma) dissolved in sunflower seed oil (Sigma) was intraperitoneally administered according to the schematic illustration described in the paper.

### Establishment of GICs with stem-like features

Single-cell tumor suspensions were plated to grow neurospheres at a density of roughly 5 × 10^5^ cells/well in 6-well Ultra-Low Attachment Multiple Well Plate (CLS3471, CORNING Costar) in the NBE medium (neurobasal medium containing N-2 and B-27 supplements, EGF, bFGF, and penicillin-streptomycin) as described. We used short-term passage cells (less than five passages in culture) in all experiments.

### Intracranial allograft and xenograft implantation of GICs

Experiments were carried out using 6-week-old male Balb/c athymic nude mice or CD-1 mice (HNSJA). A total of 1 × 10^5^ GICs were injected intracranially using a stereotactic device (RWD) and a Hamilton syringe at a depth of 2.5 mm into the right cerebral hemisphere (2 mm posterior and 1.5 mm lateral to the bregma). Animals were sacrificed 4–8 weeks post-surgery.

### Tissue processing

Mice were deeply anesthetized with 0.7% pentobarbital sodium and transcardially perfused with 4% paraformaldehyde in PBS. Brains were collected and post fixed in 4% paraformaldehyde in PBS overnight, followed by dehydration in 20% sucrose in PBS until submerging to the bottom (24–48 h). Brains were embedded in optimal cutting temperature compound (O.C.T. Compound, SAKURA) and then sliced coronally at 20 μm equidistant intervals using a cryostat microtome (CM1950, Leica).

### Flow cytometry

GBM regions from Luc positive mice were acutely dissected under fluorescent microscope and enzymatically dissociated into single-cell suspensions using the Papain Dissociation system (Worthington Biochemical). The same areas from the control mice were parallelly dissociated into single-cell suspensions to set up voltage parameters and all gates. Flow cytometry was performed on a BD LSRFortessa X-20 Cell Analyzer or a Beckman Cytoflex Cell Analyzer. Cell sorting was performed on a BD FACSAria III Cell Sorter. Every experimental group used 3 million cells for flow cytometry analyses.

### Human GBM specimens

Human GBM samples and derived GBM stem-like cells were previously reported (Zhong et al., 2016; Li et al., 2017). Their acquisition was in accordance with ethic guidelines of Wuhan Union Hospital and Shanghai Tenth People’s Hospital in accordance with institution-approved protocols. Specimens were examined by neuropathologists to verify tumor type and grade.

### Immunohistochemistry, immunofluorescence, and Western blot analysis

Tissue sections or adherent cultured cells were incubated for one hour at room temperature in blocking solution (50 mmol/L Tris-HCl, pH 8.0, 0.1 mol/L NaCl, 0.1% Triton X-100, 3% NGS, 0.1% BSA) prior to overnight primary antibody incubation (4°C). Primary antibodies for immunofluorescence studies were: SOX2 (1:1000 dilution, Ab5603, Millipore), Ki67 (1:500 dilution, ab15580, Abcam), BrdU (1:500 dilution, ab6326, Abcam), NeuN (1:500 dilution, ab177487, Abcam), TUJ1 (1:200 dilution, T8660, Sigma-Aldrich), GFAP (1:500 dilution, Z0334, Agilent), MBP (1:300 dilution, SMI-99P, Covance), OLIG2 (1:200 dilution, AB9610, Millipore), OLIG2 (1:50 dilution, MABN50, Millipore), IBA1 (1:200 dilution, ab5076, Abcam), CD31 (1:100 dilution, ab24590, Abcam), ACTA2 (1:400 dilution, ab5694, Abcam), GFP (1:2000 dilution, ab13970, Abcam), CD31 (1:300 dilution, AF3628-SP, R&D), CD146 (1:100 dilution, 134701, BioLegend). We used immunofluorescence staining with Alexa Fluor 488, 555, or 647 (Life Technologies) and biotin-streptavidin-Alexa Fluor-conjugated secondary antibodies (Jackson ImmunoResearch), as well as horseradish peroxidase-based Vectastain ABC Kit (Vector Laboratories). Image acquisitions were performed using a Zeiss LSM880 confocal microscope and image editing done using ZEN, Photoshop or ImageJ.

### Bulk RNA isolation and sequencing

RNA isolation was performed using the RNAiso Plus (TAKARA) according to the manufacturer’s instructions. RNA quality was evaluated spectrophotometrically, and the quality was assessed with the Agilent 2100 Bioanalyzer (Agilent Technologies). All samples showed RNA integrity of >7.5. RNA-seq libraries were prepared using the NEBNext Ultra II RNA Library Prep Kit for Illumina (New England Biolabs, no. E7775). Once prepared, indexed complementary DNA (cDNA) libraries were pooled in equimolar amounts and sequenced with paired-end reads on an Illumina NovaSeq 6000 (Illumina). For NPC^TKO^ tumor samples, GBM bulks were dissociated into single-cell suspensions as described above, EGFP^+^ or DsRed^+^ GBM cells were sorted by FACS followed by RNA isolation and subsequent RNA-seq. For NSC^HRas-shp53^ tumor samples, GBM bulks were directly used to isolate RNA for RNA-seq.

### Bulk RNA-seq analysis

Quality control of bulk RNA-seq data was performed using FastQC (v0.11.9). Adapter and low-quality bases were trimmed by Cutadapt (v3.2). Clean data were mapped to mouse reference genome (UCSC mm10) by TopHat (v2.1.1) with default params. Cufflinks (v2.2.1) package calculated the gene expression level and normalized by fragments per kilobase of exon model per million mapped fragments. Single-sample GSEA has been described elsewhere (Verhaak et al., 2010). ssGSEA calculated separate enrichment scores between each sample and the specified gene sets that were download from published reference data. An enrichment score calculated by R package GSVA [v1.38.2, gsea (method = “ssgsea”, ssgsea.norm = TRUE)]. A positive score represents a high ranking of up-genes in the signature, and low ranking of down-genes in the signature. A negative value does not indicate the opposite, but rather a lack of effect. For the experiment described in Fig. S1, gene sets for related neural lineage clusters (Fig. S1N; Cahoy et al., 2008), TCGA-GBM subtypes (Fig. S1M; Verhaak et al., 2010), lineage-specific GBM subtypes (Fig. S1O; Wang et al., 2020) were downloaded and processed as described above.

### scRNA-seq data generation and processing

Designated cells were sorted into PBS following the 10× Genomics protocol. The cell preparation time before loading onto the 10× Chromium controller was <2 h. Cell viability and counting were evaluated with trypan blue by microscopy, and samples with viabilities >70% were used for sequencing. Libraries were constructed using the Single Cell 3ʹ Library Kit V2 (10× Genomics). Transcriptome profiles of individual cells were determined by 10× Genomics-based droplet sequencing. Once prepared, indexed cDNA libraries were sequenced with paired-end reads on an Illumina NovaSeq 6000 (Illumina). The sequencing depth was 30 M per cell.

### scRNA-seq data processing

The quality of sequencing reads was evaluated using FastQC (v0.11.9). Cell Ranger (v4.0.0) was used to align the sequencing reads (fastq) to the mm10 mouse transcriptome and quantify the expression of transcripts in each cell. This pipeline resulted in a gene expression matrix for each sample, which records the number of unique molecular identifiers (UMIs) for each gene associated with each cell barcode. Unless otherwise stated, all downstream analyses were implemented using R 3.6.0 and the package Seurat (v3.1.0). The percentage of mitochondrial and red blood cells were calculated and filtered to retain only higher-quality cells (red blood cells <5%). The feature count matrix was normalized and scaled with NormalizeData (normalization.method = “LogNormalize”, scale.factor = 10,000) and ScaleData function. We performed doublet prediction on the clustered data using Doublet Finder. For the experiment described in Fig. 4, cells from two samples were pooled and analyzed together. After rigorous quality control, in NPC^TKO^ #1, we obtained 11,722 high-quality cells with a median gene number—4,450 genes, resulting in a total of 19,878 mouse genes detected in all cells. In NPC^TKO^ #2, we obtained 13,945 high-quality cells with a median gene number—3,402 genes, resulting in a total of 20,038 mouse genes detected in all cells.

### Dimension reduction

Dimension reduction was performed at three stages of the analysis: the selection of variable genes, PCA, and UMAP. The FindVariableGenes function was applied to select highly variable genes covering most of the biological information contained in the whole transcriptome. Then, the variable genes were used for PCA implemented with the RunPCA function. Next, we selected principal components 1–20 (for total cells) as input and performed the RunUMAP function to obtain bidimensional coordinates for each cell. We then clustered the cells using FindClusters (resolution = 0.6) function.

### Determination of cell-type identity

For each cell type, we used multiple cell type-specific/enriched marker genes that have been previously described in the literature to determine cell-type identity. For ECs: *Pecam1*, *Tek*, and *Cldn5* (Vanlandewijck et al., 2018). For mural cells: *Kcnj8*, *Pdgfrb*, *Acta2*, and *Tbx18* (Vanlandewijck et al., 2018). For T cells: *Cd2*, *Cd3d*, *Cd3e*, and *Cd3g*. For TAMs: *Cd14*, *Tmem119*, and *Aif1*. For OLGs: *Cldn11*, *Mag*, *Mbp*, and *Klk6* (Brown et al., 2016).

### CNV inference from single-cell data using inferCNV

CNVs were estimated by sorting the analyzed genes by their chromosomal location and applying a moving average to the relative expression values, with a sliding window of 100 genes within each chromosome by inferCNV. For mouse data in Figs. 4 and S6, T cells were used to define the baseline of normal karyotype as the non-malignant cell types, such that their average CNV value was subtracted from all cells. Using hierarchical clustering of the rest single cells, three groups with the most significant and concordant CNV profiles were identified, and were named as Group A/B/C cells, respectively. Percentages of Group A/B/C cells in each Seurat clusters were presented (Fig. 4F and 4G). Then, we applied the “two CNA-based measures” to score each cell as previously reported (Neftel et al., 2019) to compare the lineage relationship of target Seurat clusters with Group A/B/C cells, respectively. “CNV signal” reflects the overall extent of CNVs, defined as the mean of the squares of CNV values across chosen chromosomes containing significant and concordant CNV profiles. In NPC^TKO^ #1, we selected chromosome 2, 3, 5, 8, 11, 12, 13, 15 as CNV profile for Group A cells; Chr 5, 11, 12, 13 for Group B cells; Chr 6, 13, 18, 19 for Group C cells. In NPC^TKO^ #2, we selected chromosome 2, 8, 9, 10, 11, 14, 15, 17, 19 as CNV profile for Group A cells; Chr 2, 9, 10, 11, 14, 15, 17, 19 for Group B cells; Chr 7, 18 for Group C cells. “CNV correlation” refers to the correlation between the chosen CNV profile of each cell and the average CNV profile of Group A/B/C cells, respectively. For human data in Figs. 5 and S7, T cells, TAMs, and OLGs were used to define the baseline of normal karyotype as the non-malignant cell types (Neftel et al., 2019). In SF11247, we selected chromosome 7, 10, 13, 19 as CNV profile for Group A cells; Chr 3, 7, 13 for Group B cells. In SF11285, we selected all chromosomes across the genome as CNV profile for Group A cells; Chr 7, 8, 12, 18, 19 for Group B cells.

### CopyKAT

To distinguish malignant cells from non-malignant cells in human GBM samples, genome-wide aneuploidy in single cells were identified at 5 MB resolution to separate tumor cells from normal cells by copyKAT (V1.0.8.) using integrative Bayesian approaches. For experiments described in Fig. 5, raw gene expression matrixes of two GBM samples were prepared to run copyKAT, respectively. The parameter “ngene.chr=1” was applied to keep as many cells as possible.

### Measurements and statistics

To quantify the percentage of marker-positive tumor cells, numbers of NeuN-, MBP-, GFAP-, OLIG2-, or SOX2-expressing tumor cells were counted in tumor cores, then divided by numbers of total tumor cells (EGFP or DsRed positive cells). To quantify the percentage of BrdU^+^ or Ki67^+^ ECs or mural cells, double-positive cells (BrdU^+^tdTomato^+^ or Ki67^+^tdTomato^+^) were counted in both tumor and non-tumor sites, then divided by numbers of total ECs or mural cells (>1000 cells). Vessel area and numbers were analyzed using glioma brains from three NPC^TKO^ and three NSC^HRas-shp53^ model generated in *Tbx18*^*CreERT2*/+^;*Ai14* mice. Fluorescence sections were scanned with Leica THUNDER Imager Tissue Scanner. For each mouse brain, two images were cropped at the tumor core (without necrosis) and at the matched region of the contralateral non-tumor parenchyma, respectively. Average number of vessels per mm^2^ and average total vascular area per field were measured with the ImageJ and analyzed with Graphpad Prism. OLIG2^+^ cells density was analyzed using glioma brains from eight NPC^TKO^ and seven NSC^HRas-shp53^ model generated in *Tek-Cre*;*Ai14* and *Tbx18*^*CreERT2*/+^;*Ai14* mice. Immunohistochemistry sections were scanned with the Leica Aperio VERSA 8 Brightfield, Fluorescence & FISH Digital Pathology Scanner. The number of OLIG2^+^ cells per mm^2^ in each mouse brain tumor core was measured with the ImageJ and analyzed with Graphpad Prism. All grouped data are presented as mean ± SEM. Statistical tests were calculated on the GraphPad Prism software (version 8.4.3). The statistical significance of a single comparison on continuous data was performed using Student’s two-tailed unpaired *t*-test with Welch’s correction when required (non-equal variances) or the Mann–Whitney non-parametric test when data did not fit a normal distribution (assessed by Shapiro–Wilk normality test). For multiple comparison of Ki67^+^tdTomato^+^ in Fig. 6B, one-way analysis of variance (ANOVA) followed by Tukey’s *post hoc* test was performed. For multiple comparison of vascular density in Fig. 1I and 1J, we used two-way ANOVA, after checking that our data fitted to a normal distribution (assessed by Shapiro–Wilk normality test). Significant difference is indicated by a *P*-value less than 0.05 (*P < 0.05, **P < 0.01, ***P < 0.001, and ****P < 0.0001).

## Supplementary Material

pwac006_suppl_Supplementary_Material

pwac006_suppl_Supplementary_Table_S1

pwac006_suppl_Supplementary_Table_S2

pwac006_suppl_Supplementary_Table_S3

pwac006_suppl_Supplementary_Table_S4

pwac006_suppl_Supplementary_Table_S5

pwac006_suppl_Supplementary_Table_S6

pwac006_suppl_Supplementary_Video_S1

pwac006_suppl_Supplementary_Video_S2

## Data Availability

The accession number for the RNA-seq and single-cell transcriptome data reported in this paper is GEO accession number GSE189541 and GSE190154. Human data used in Figs. 5 and S7 have been deposited as PRJNA579593 from a published study (Bhaduri et al., 2020).
