# Supplementary material for: Resolving the lineage relationship between malignant cells and vascular cells in glioblastomas
Source: Protein Cell. 2022 May 18;14(2):105–22. doi: 10.1093/procel/pwac006 (PMC10019576; doi:10.1093/procel/pwac006)
Supplement: pwac006_suppl_Supplementary_Material [file pwac006_suppl_supplementary_material.pdf]

# Supplemental materials

## Resolving the lineage relationship between malignant cells and vascular cells in glioblastomas

**Fangyu Wang[1,4], Xuan Liu[1,4], Shaowen Li[1], Chen Zhao[1], Yumei Sun[1], Kuan Tian[1], Junbao Wang[1], Wei Li[1], Lichao Xu[1], Jing Jing[1], Juan Wang[3], Sylvia M Evans[2], Zhiqiang Li[1,*], Ying Liu[1,*], Yan Zhou[1,*]**

**Video S1.** 3-D image reconstruction of Z-stack images of the section of a *Tbx18*$^{H2B\text{-}GFP/+}$ glioblastoma brain immuno-labelled with ACTA2 (white) showing spatial relationship between tumor cells (DsRed, red) and mural cells (nuclear GFP and cytosolic ACTA2+). Twenty consecutive confocal images (1 μm deep per image) were merged and reconstructed.

**Video S2.** 3-D image reconstruction of Z-stack images of the section of a *Tek-Cre*;*Ai14* glioblastoma brain immuno-labelled with ACTA2 showing spatial relationship between tumor cells (EGFP, green), ECs (tdTomato, red) and mural cells (ACTA2, white). Fourteen consecutive confocal images (1 μm deep per image) were merged and reconstructed.

**Table S1** (Related to Figure 4). Cluster markers and cell type interpretations for $NPC^{TKO}$ GBMs

**Table S2** (Related to Figure 4). CNV correlations and scores for Group A/B/C cells in $NPC^{TKO}$ #1

**Table S3** (Related to Figure 4). CNV correlations and scores for Group A/B/C cells in $NPC^{TKO}$ #2

**Table S4** (Related to Figure 5). Cluster markers and cell type interpretations for human GBM SF11247

**Table S5** (Related to Figure 5). Cluster markers and cell type interpretations for human GBM SF11285

**Table S6** (Related to Figure 5). Assigning single cells of human GBM SF11247 and SF11285 into aneuploid and diploid cells using the copyKAT algorithm.

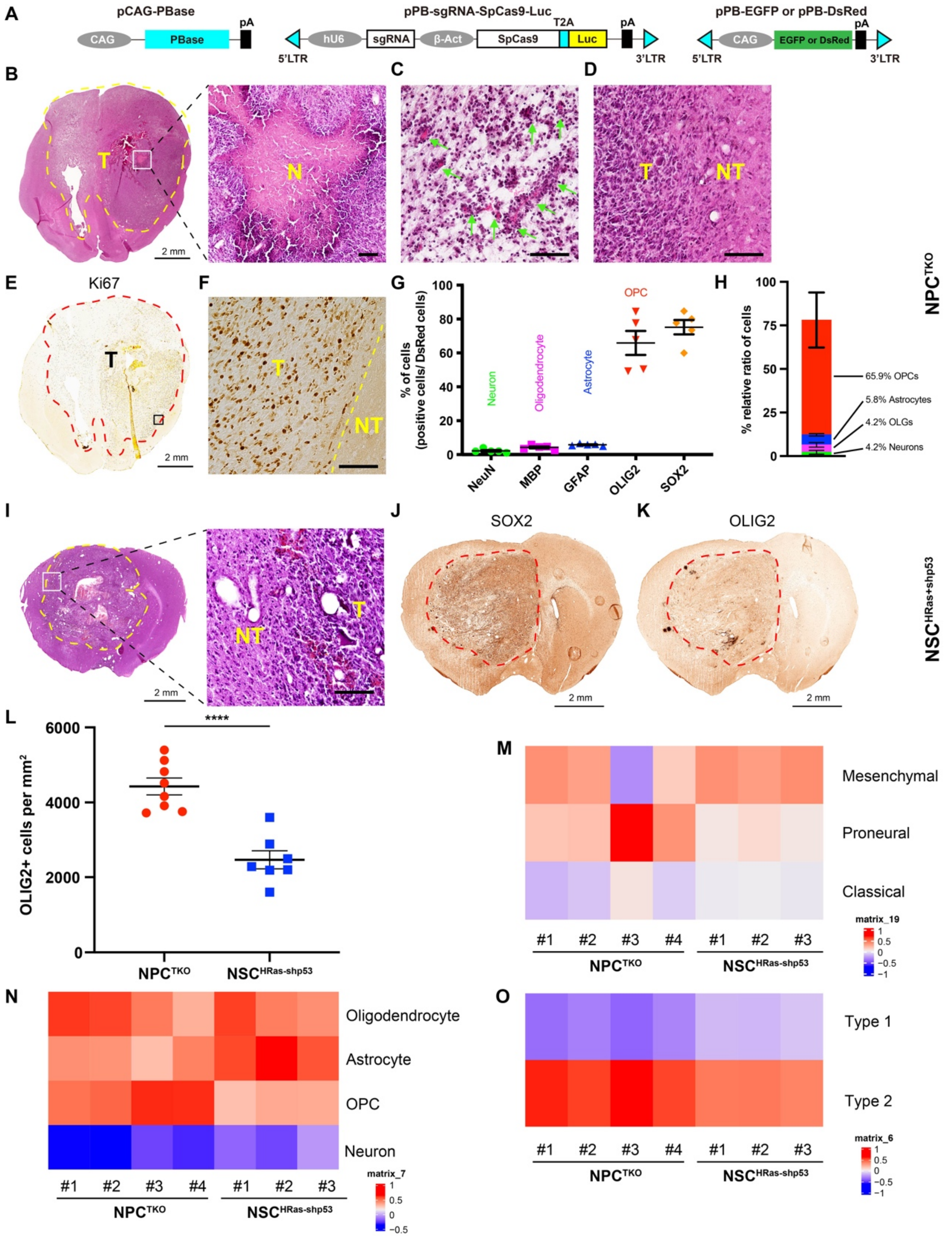

**Figure S1 (related to Figure 1). NPC$^{TKO}$ and NSC$^{HRas\text{-}shp53}$ GBM models recapitulate key histological and molecular features of human GBMs.**

(**A**) Schematic illustrations showing electroporation vectors for generating NPC$^{TKO}$ GBMs. (**B-D**) H&E staining of NPC$^{TKO}$ glioblastoma brain sections showing necrotic (N) tumor core (**B**). Scale bars, 2 mm (left), 500 μm (right). Prominent tumor vessels (**C**, arrows) and tumor invasion (**D**). Scale bars, 100 μm. (**E-F**) Enhanced cell proliferation of NPC$^{TKO}$ GBM cells evidenced by more

Ki67+ cells in tumor (T) region compared to non-tumor (NT) region. Scale bars, 100 μm. (**G-H**) $NPC^{TKO}$ GBM brain sections were immunostained with indicated markers and quantified (n = 5 animals). (**I**) H&E staining of $NSC^{HRas\text{-}shp53}$ glioblastoma brain sections showing the border of tumor (T) region and non-tumor (NT) region, Scale bars, 100 μm. (**J-K**) $NSC^{HRas\text{-}shp53}$ glioblastoma brain sections were immunostained with SOX2 (**J**) and OLIG2 (**K**). (**L**) The comparison of OLIG2+ cell densities between $NPC^{TKO}$ and $NSC^{HRas\text{-}shp53}$ GBM brains. n = 8 for $NPC^{TKO}$ brains and n = 7 for $NSC^{HRas\text{-}shp53}$ brains. (**M-O**) Transcriptome features of $NPC^{TKO}$ and $NSC^{HRas\text{-}shp53}$ GBMs were compared with those of public datasets. Data are mean ± s.e.m. Statistical significance was determined using an unpaired two-tailed Student's *t*-test (**L**), ****$P$<0.0001.

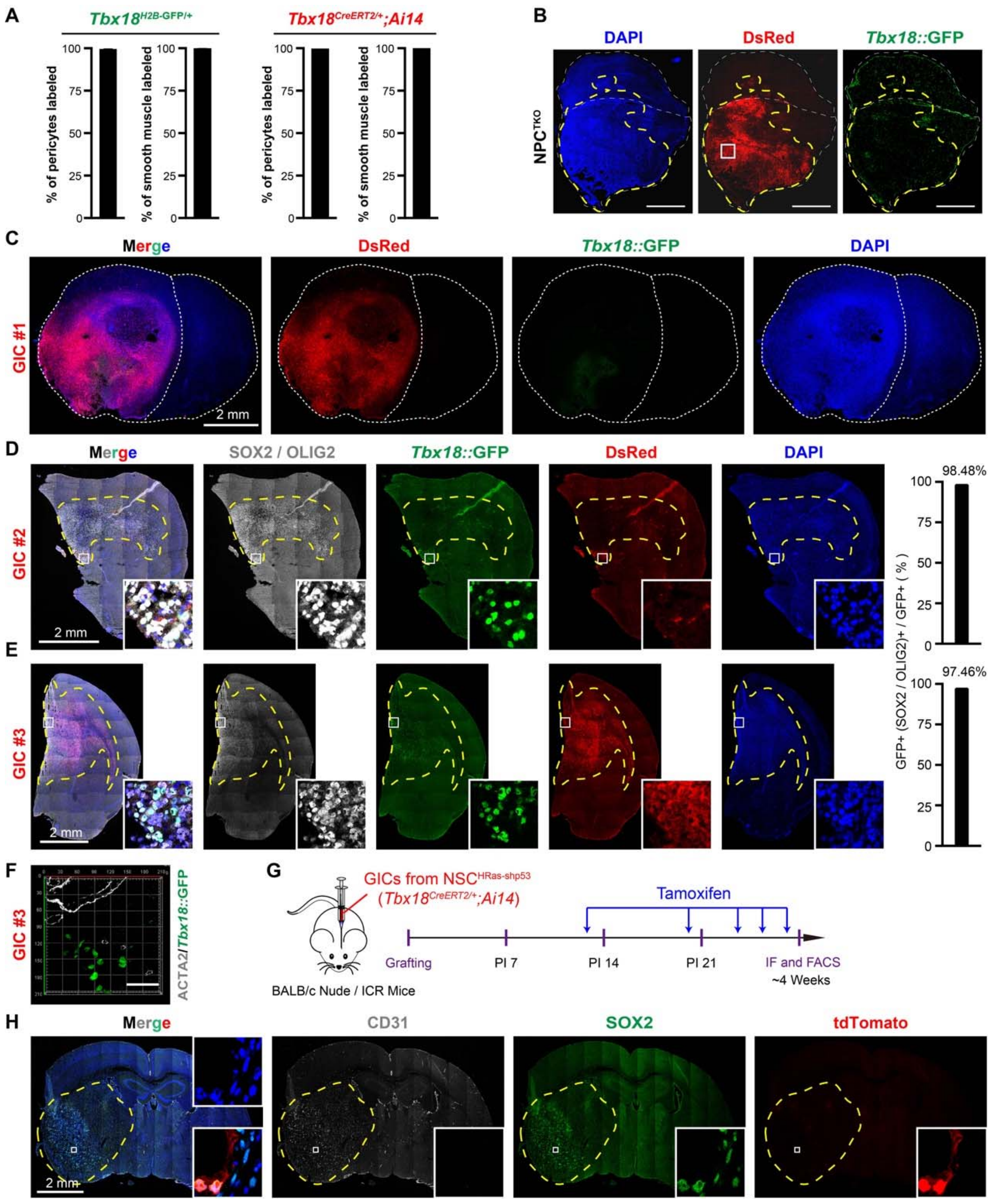


**Figure S2 (related to Figure 2). GBM cells hardly gave rise to mural cells, but could promiscuously express *Tbx18* upon allografting.** (**A**) Bar plots showing percentages of pericytes (marker+CD146+PDGFRB+/ CD146+PDGFRB+) and vascular smooth muscle cells (vSMCs, marker+ACTA2+/ACTA2+) labelled by *Tbx18::*GFP (left) or *Tbx18*-driven tdTomato (right) in normal mouse brains. (**B**) The coronal section of a $NPC^{TKO}$ ($Tbx18^{H2B\text{-}GFP/+}$) mouse brain with mural cells labelled with nuclear H2B-GFP and GBM cells labelled with DsRed. Scale bars, 2 mm. Boxed area was enlarged in **Figure 2B**. (**C-F**) BALB/c nude mice were respectively

intracranially allografted with indicated GIC lines derived from NPC$^{TKO}$ (*Tbx18*$^{H2B\text{-}GFP/+}$) GBMs. Representative immunofluorescent images showing expressions of *Tbx18::*H2B-GFP, allografted DsRed-expressing GICs, and SOX2/OLIG2. In (**D**) and (**E)**, percentages of GFP cells co-expressing SOX2/OLIG2 were shown as bar graphs on the right. Scale bars, 2 mm. Representative Z-stack images showing expressions of *Tbx18::*H2B-GFP and spatial relationships of ACTA2+ mural cells (**F**). Scale bars, 60 µm. (**G-H**) Schematic illustration showing GICs derived from a NSC$^{HRas\text{-}shp53}$ (*Tbx18*$^{CreERT2/+}$*;Ai14*) GBM sample were implanted into brains of BALB/c nude and ICR mice. Tamoxifen was injected once per week for the first two weeks and three times in the third week (**G**). Representative images showing immunofluorescent staining of brain sections using indicated antibodies (**H**).

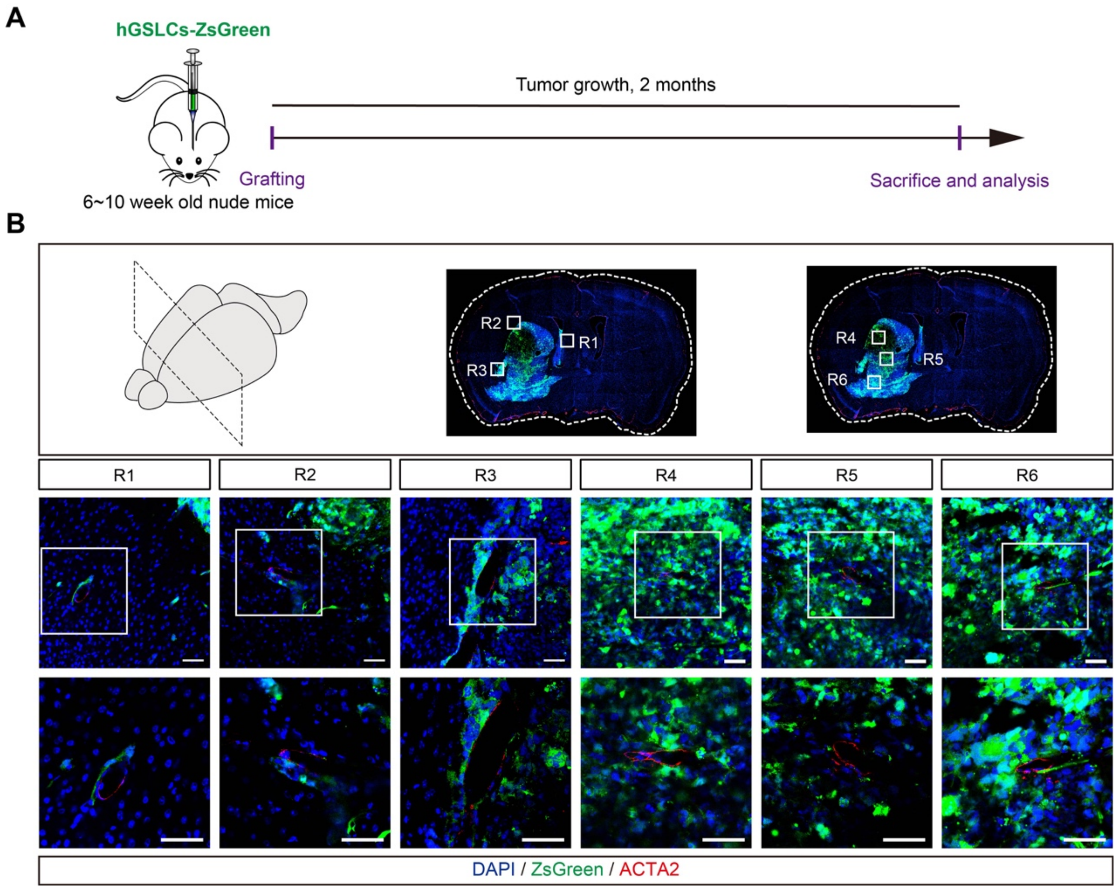


**Figure S3 (related to Figure 2). Human GBM cells did not give rise to mural cells upon intracranial transplantation.** (**A**) Schematic illustration showing ZsGreen-labeled GBM stem-like cells derived from a human GBM sample were implanted into brains of BALB/c nude mice. (**B**) Representative images showing spatial relationships of ZsGreen-labeled human GBM cells and ACTA2-expressing mural cells. Boxed regions of brain sections on the top were enlarged in middle and bottom panels. Scale bars, 50 μm.

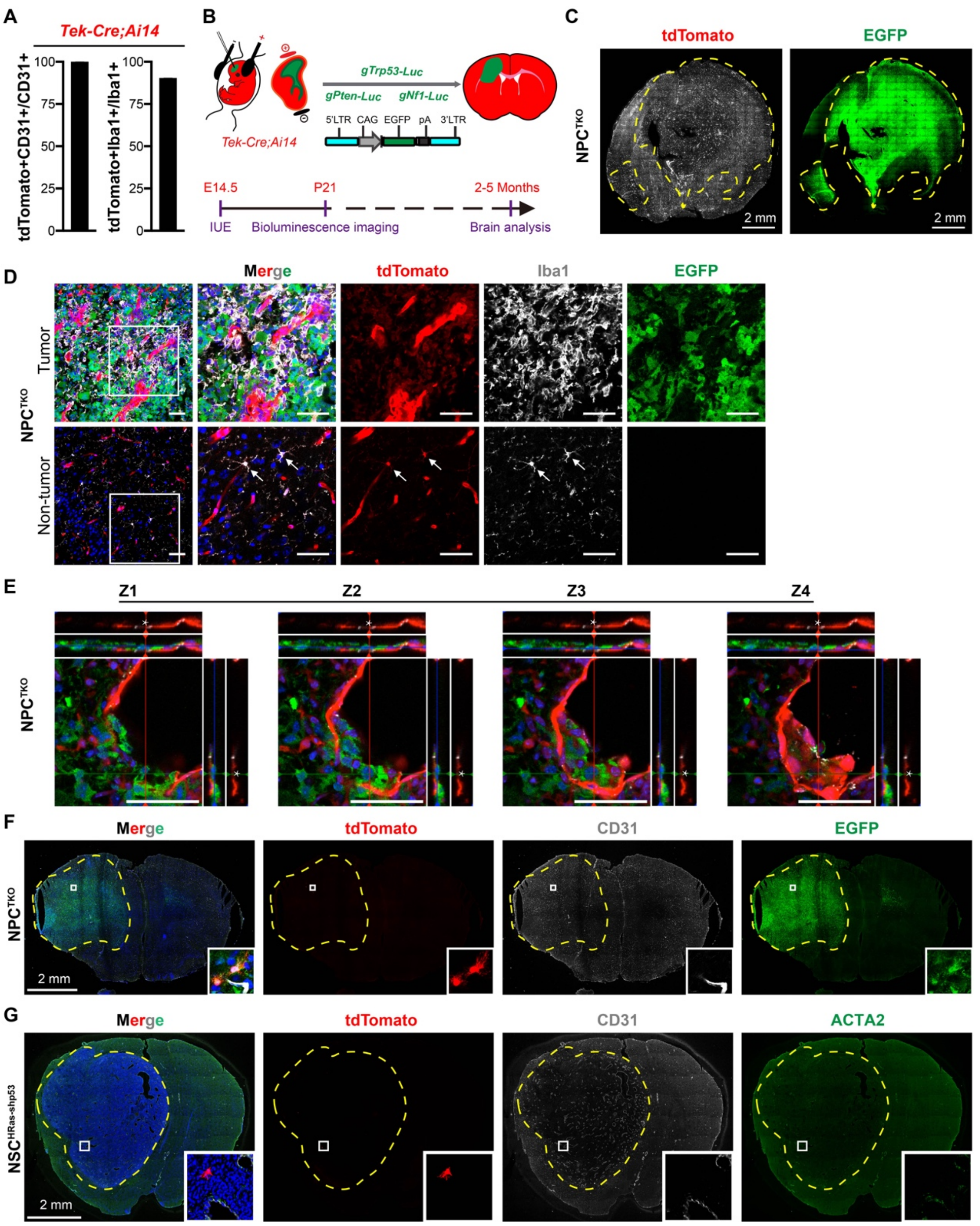


**Figure S4 (related to Figure 3). ECs and glioblastoma cells are spatially distinct cell population.** (**A**) Bar plots showing percentages of ECs (left) and TAMs (right) labelled by *Tek*-driven tdTomato in wildtype mouse brains. (**B**) Top: the schematic illustration showing *in vivo* gliomagenesis of NPC$^{TKO}$ GBMs in *Tek-Cre*;*Ai14* mice where transformed glioblastoma cells were lineage-labelled with stably-expressed EGFP. Bottom: the timeline for *in vivo* gliomagenesis by *in utero* electroporation (IUE), live tumor detection and phenotypic analyses. (**C**) The coronal section of a NPC$^{TKO}$ (*Tek-Cre*;*Ai14*) mouse brain with ECs and

TAMs labelled with tdTomato and GBM cells labelled with EGFP. Scale bars, 2 mm. (**D**) Representative GBM (top) and non-tumor (bottom) brain sections were immunostained with TAMs using the anti-Iba1 antibody. Boxed areas were enlarged on right panels. Scale bars, 50 μm. **(E**) Four consecutive Z-stack images showing EGFP-labelled GBM cells have close proximity to tumor vasculatures without erosion of the vascular wall. Scale bars, 50 μm. (**F-G**) BALB/c nude mice were intracranially allografted with GICs derived from $NPC^{TKO}$ (**F**) and $NSC^{HRas\text{-}shp53}$ (**G**) *Tek-Cre*;*Ai14* GBMs. Representative images showing immunofluorescent staining of brain sections using indicated antibodies.

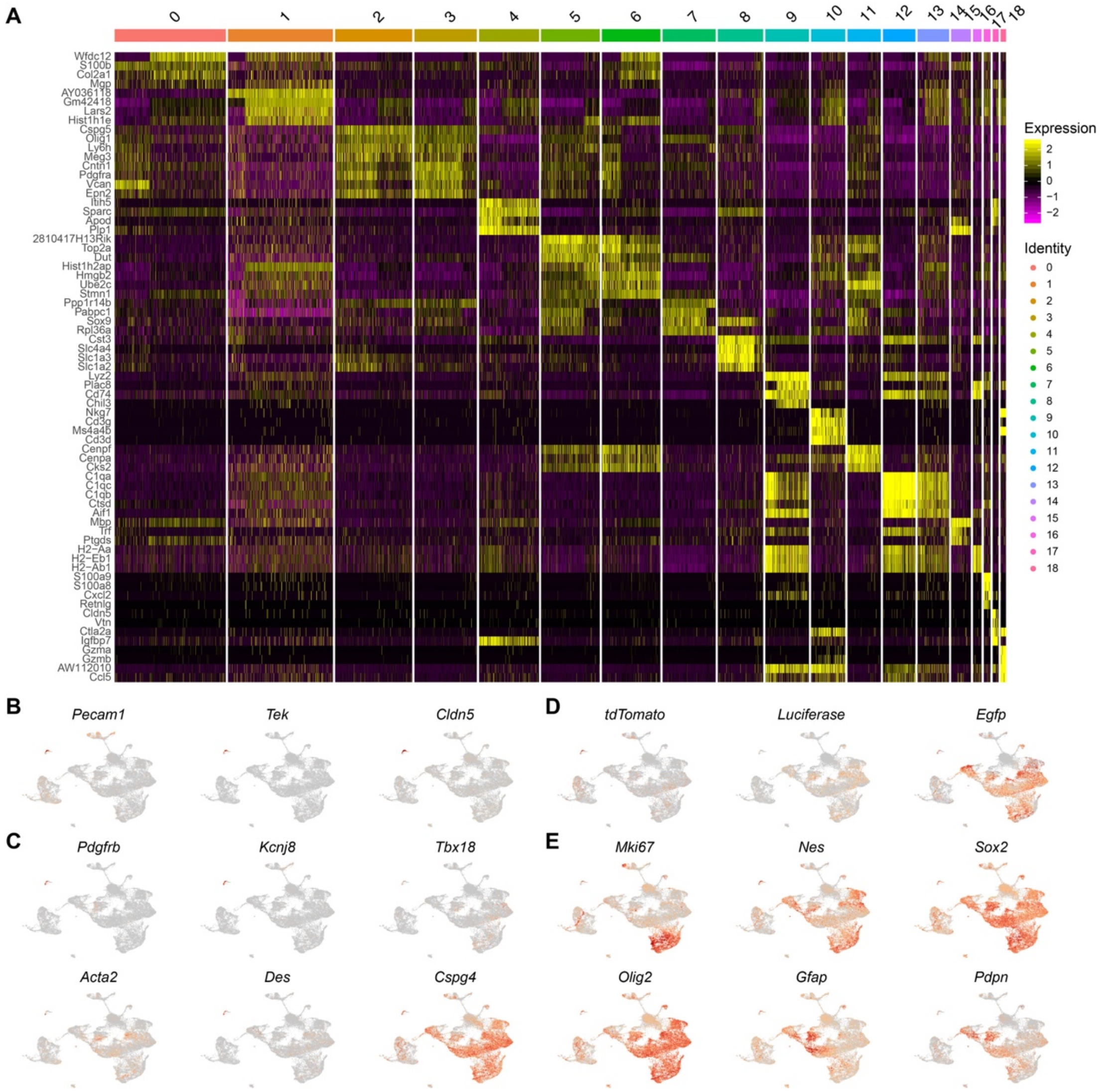


**Figure S5 (related to Figure 4). Marker expressions and distributions of NPC$^{TKO}$ GBMs.** (**A**) Heatmap of NPC$^{TKO}$ GBM cells ordered as UMAP. Columns, individual cells; rows, top 4 markers for each UMAP cluster. (**B-E**) Expression and distribution of indicated markers in NPC$^{TKO}$ GBMs on UMAP plots.

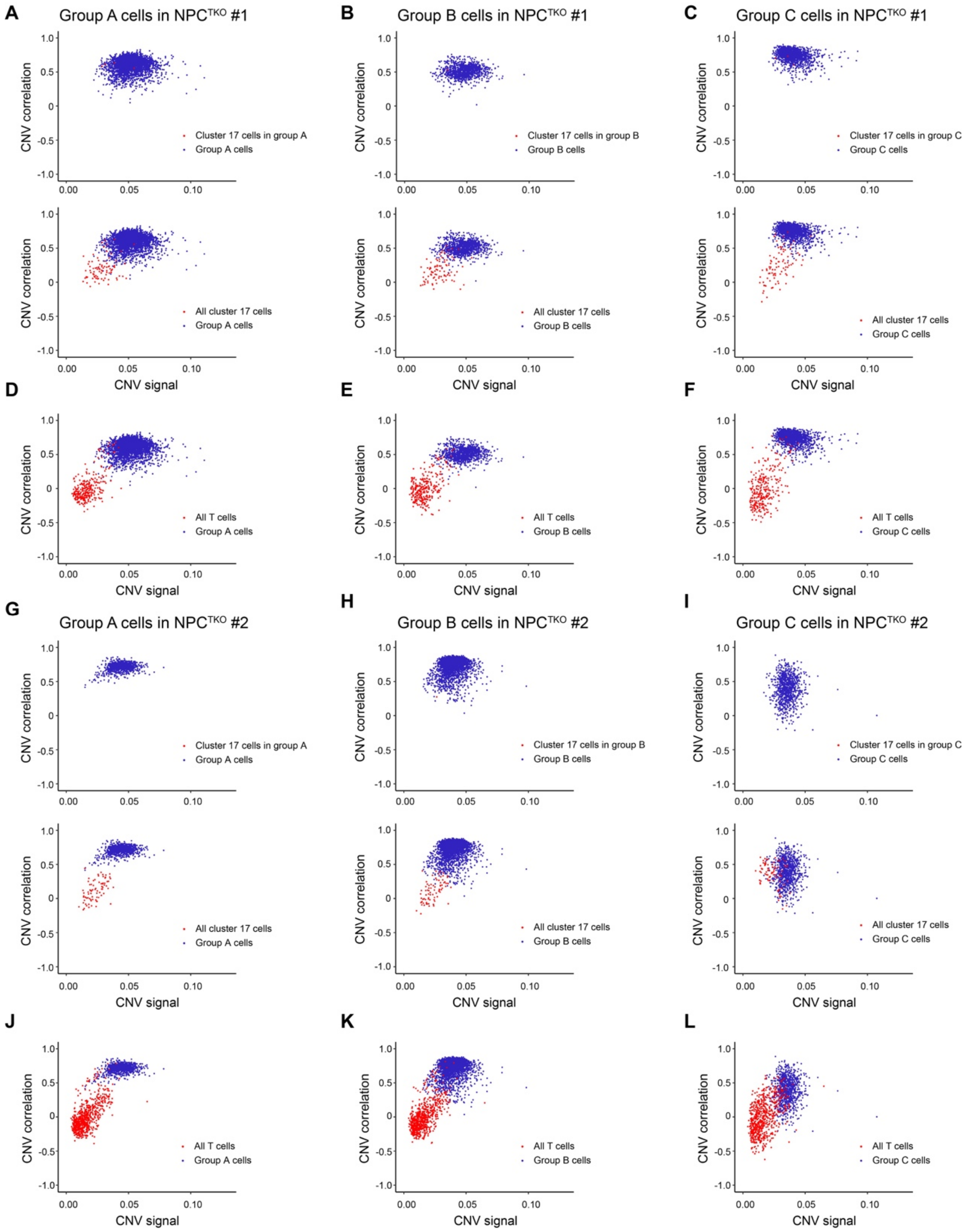


**Figure S6 (related to Figure 4). Plots depicting CNV signals and correlations of vascular cells (cluster 17), T cells and different groups of malignant cells.** ‘CNV signal’ reflects the overall extent of CNVs of each cell, defined as the mean of the squares of CNV values across the genome. ‘CNV correlation’ refers to the correlation between the CNV profile of each cell and the average CNV profile of all malignant cells from the corresponding tumor.

Group A, B, C malignant cells were indicated in **Figure 4D** and **4E**.

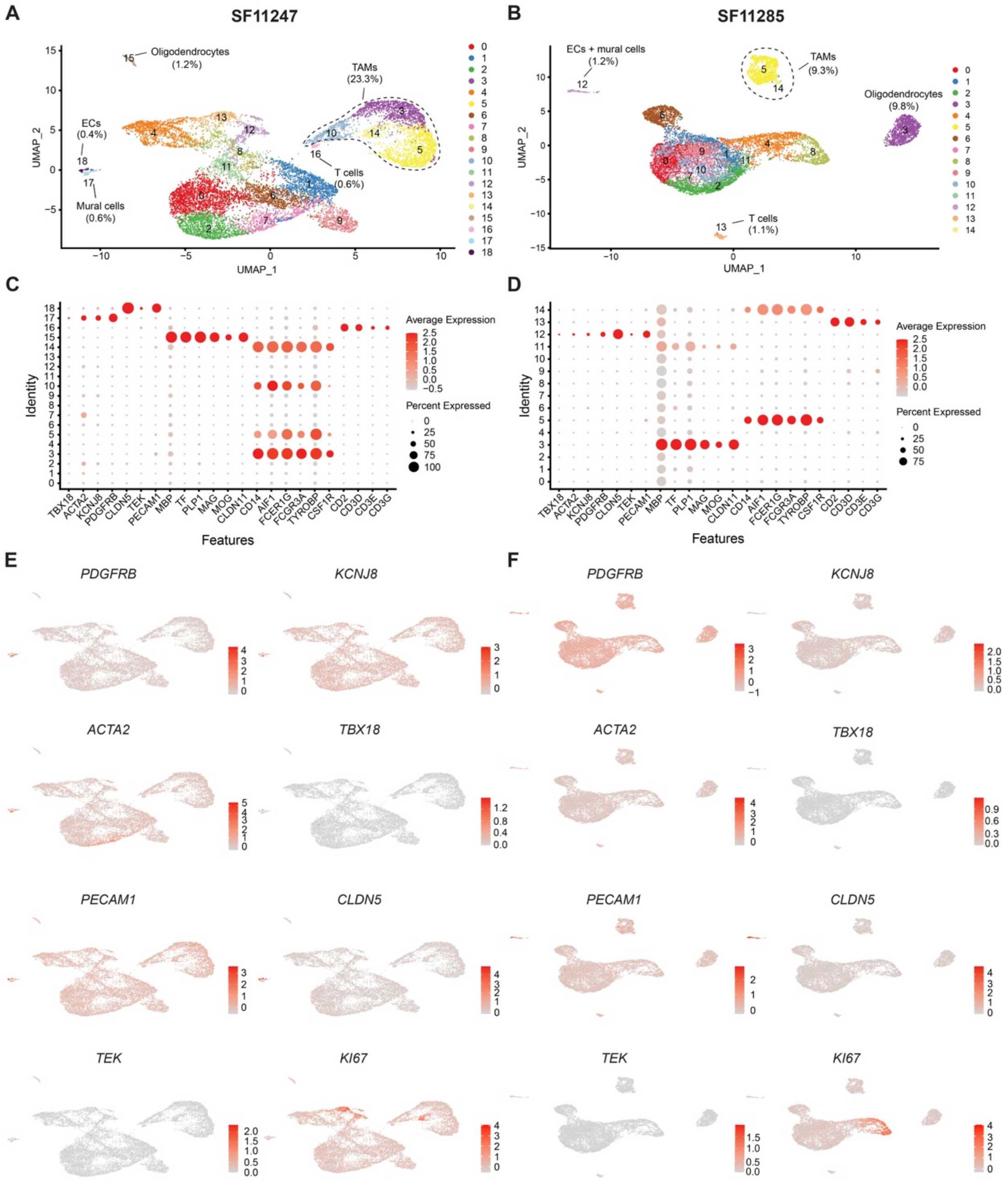


**Figure S7 (related to Figure 5). Cell clustering and marker expressions of human GBM samples - SF11247 (A, C and E) and SF11285 (B, D and F).**

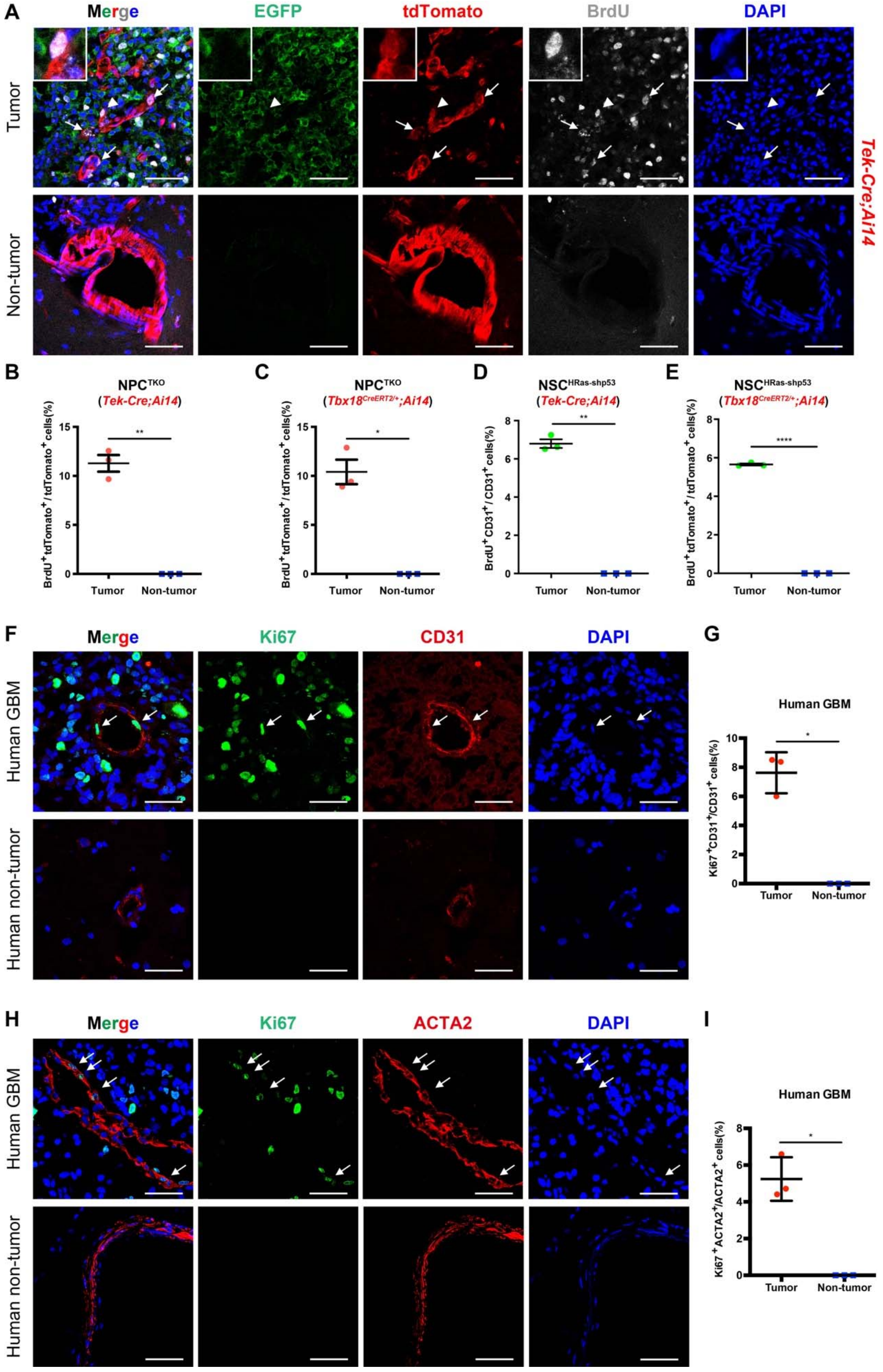


**Figure S8 (related to Figure 6). ECs and mural cells self-propagate during gliomagenesis.**

(**A**) Top: representative immunofluorescent images showing GBM brain sections from a *Tek-Cre*;*Ai14* mouse were immunostained for BrdU. BrdU+ ECs were indicated with arrows, with a dividing EC enlarged in the left corners (arrowhead). Bottom: representative immunofluorescent images at a non-tumor site. (**B-E**) Percentages of BrdU+tdTomato+/tdTomato+ cells in *Tek-Cre*;*Ai14* (**B**) and in $Tbx18^{CreERT2}$;*Ai14* (**C** and **E**), of BrdU+CD31+/CD31+ cells in *Tek-Cre*;*Ai14* (**D**) GBM brains were quantified respectively. (**B**-**C**) and (**D**-**E**) are data collected from $NPC^{TKO}$ and $NSC^{HRas\text{-}shp53}$ brains respectively. n = 3 for tumor and non-tumor tissues. (**F-I**) Representative immunofluorescent images for human GBM (top) and non-tumor (bottom) tissues immunostained with Ki67, CD31 (**F**) and ACTA2 (**H**). Scale bars, 50 μm. Percentages of Ki67+CD31+/CD31+ ECs and Ki67+ACTA2+/ACTA2+ mural cells were quantified in (**G**) and (**I**) respectively. n = 3 for tumor and non-tumor tissues. Data are mean ± s.e.m. Statistical significance was determined using an unpaired two-tailed Student's *t*-test (**B**, **C**, **D**, **E**, **G**, **I**), **P*<0.05; ***P*<0.01; ****P*<0.001; *****P*<0.0001.
